# Physiological effects of over-expressing compartment-specific components of the protein folding machinery in xylose-fermenting *Saccharomyces cerevisiae*

**DOI:** 10.1186/1472-6750-14-28

**Published:** 2014-04-23

**Authors:** Basti Bergdahl, Marie F Gorwa-Grauslund, Ed WJ van Niel

**Affiliations:** 1 Division of Applied Microbiology, Department of Chemistry, Lund University, P.O. Box 124, Lund SE-22100, Sweden

**Keywords:** *Saccharomyces cerevisiae*, Xylose fermentation, Protein folding, Transcription analysis, Fumarate reductase, Endoplasmic reticulum, Disulphide bond formation, FRD1, OSM1, ERO1

## Abstract

**Background:**

Efficient utilization of both glucose and xylose is necessary for a competitive ethanol production from lignocellulosic materials. Although many advances have been made in the development of xylose-fermenting strains of *Saccharomyces cerevisiae*, the productivity remains much lower compared to glucose. Previous transcriptional analyses of recombinant xylose-fermenting strains have mainly focused on central carbon metabolism. Very little attention has been given to other fundamental cellular processes such as the folding of proteins. Analysis of previously measured transcript levels in a recombinant XR/XDH-strain showed a wide down-regulation of genes targeted by the unfolded protein response during xylose fermentation. Under anaerobic conditions the folding of proteins is directly connected with fumarate metabolism and requires two essential enzymes: FADH_2_-dependent fumarate reductase (FR) and Ero1p. In this study we tested whether these enzymes impair the protein folding process causing the very slow growth of recombinant yeast strains on xylose under anaerobic conditions.

**Results:**

Four strains over-expressing the cytosolic (*FRD1*) or mitochondrial (*OSM1*) FR genes and *ERO1* in different combinations were constructed. The growth and fermentation performance was evaluated in defined medium as well as in a complex medium containing glucose and xylose. Over-expression of *FRD1*, alone or in combination with *ERO1*, did not have any significant effect on xylose fermentation in any medium used. Over-expression of *OSM1*, on the other hand, led to a diversion of carbon from glycerol to acetate and a decrease in growth rate by 39% in defined medium and by 25% in complex medium. Combined over-expression of *OSM1* and *ERO1* led to the same diversion of carbon from glycerol to acetate and had a stronger detrimental effect on the growth in complex medium.

**Conclusions:**

Increasing the activities of the FR enzymes and Ero1p is not sufficient to increase the anaerobic growth on xylose. So additional components of the protein folding mechanism that were identified in transcription analysis of UPR related genes may also be limiting. This includes i) the transcription factor encoded by *HAC1* ii) the activity of Pdi1p and iii) the requirement of free FAD during anaerobic growth.

## Background

The yeast *Saccharomyces cerevisiae* is one of few yeast species capable of growing under strict anaerobic conditions [1]. This trait together with a high tolerance toward inhibitory compounds, e.g. ethanol, weak acids, phenolics and furaldehydes, has made *S. cerevisiae* the organism of choice for bioethanol production [2]. The development of sustainable processes for biofuel production is an important step in the efforts to reduce greenhouse gas emissions and becoming independent of fossil fuels [3]. The utilization of lignocellulosic raw material for the production of fuel-grade ethanol is one such process currently under development [4,5]. These raw materials are generated as waste in e.g. agricultural and forestry industries and contain a large fraction of fermentable sugars. The composition of the sugar fraction varies between different materials, but the largest part often consists of glucose and xylose [4]. Efficient utilization of both these sugars is necessary for the ethanol production process to be economically feasible [6,7]. *S. cerevisiae* is well-known for its capability to ferment hexoses, especially glucose. However, *S. cerevisiae* cannot naturally utilize xylose and thus has to be genetically modified. The simultaneous expression of the *XYL1* and *XYL2* genes from *Scheffersomyces stipitis* encoding xylose reductase (XR) and xylitol dehydrogenase (XDH), respectively, is one pathway that enables xylose utilization by *S. cerevisiae*[8]. Although many advances have been made in the development of xylose-fermenting *S. cerevisiae* strains, the efficiency is still far from matching that of glucose [9,10].

**Figure 1:**
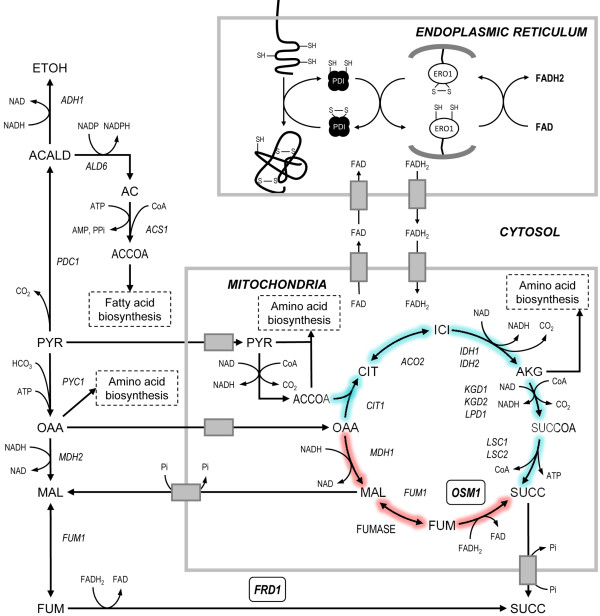
**Metabolic pathways involving Frd1p, Osm1p, Pdi1p and Ero1p.** Under anaerobic conditions the TCA cycle operates as two branches, a reductive path (red color) and an oxidative path (turquoise color). Osm1p catalyses the last step of the reductive branch of the TCA-cycle where fumarate is converted to succinate and FAD is regenerated from FADH_2_. Frd1p catalyses the same reaction in the cytosol. The regenerated FAD is used inside the ER during the maturation of secretory proteins. The Pdi1p and Ero1p enzymes mediate the oxidative folding of proteins in the ER. When a nascent polypeptide is translocated into the ER the reduced cysteines are oxidised by Pdi1p giving rise to a disulphide bridge in the protein. The reduced Pdi1p is subsequently oxidised by the membrane bound Ero1p which transfers the electrons to oxygen via covalently bound FAD. *In vitro* experiments have shown that free FAD can act as the electron acceptor in the absence of oxygen.

The ability of *S. cerevisiae* to grow under strict anaerobic conditions is dependent on the activity of fumarate reductase (FR) enzymes [11,12]. *S. cerevisiae* has two genes, *FRD1* and *OSM1*, encoding the cytosolic and mitochondrial FR enzymes, respectively [13,14]. These enzymes catalyse the reduction of fumarate to succinate while regenerating FAD from FADH_2_[15]. This is the last step of the reductive branch of the TCA-cycle where mitochondrial NAD is regenerated under anaerobic conditions (Figure 1) [16]. Single deletion of *FRD1* or *OSM1* has no effect on the ability to grow under anaerobiosis, but a double deletion mutant cannot grow under such conditions unless an external electron acceptor is supplied (e.g. oxygen, menadione or phenazine methosulfate) [11,12,17]. It has thus been proposed that FR enzymes provide the only way for yeast to regenerate the FAD/FMN prosthetic group of flavin enzymes that are required for growth under anoxia [11]. Ero1p is one such essential flavin-containing oxidase, which normally uses oxygen as a final electron acceptor for FAD regeneration. Ero1p is a thiol oxidase [18,19] that operates together with Pdi1p [20] in the endoplasmic reticulum (ER) where they take part in the maturation of secretory proteins [21]. Both proteins are essential for the formation of disulphide bonds and together they form a classical proteinaceous electron relay system in which electrons are passed from the substrate polypeptide, via Pdi1p and the membrane-bound Ero1p, to eventually react with oxygen (Figure 1) [22-24].

Approximately one third of the yeast proteome is processed in the secretory pathway which makes this mechanism indispensable for cell viability, growth and function [21]. Many of the maturation processes taking place in the ER are regulated by a mechanism referred to as the unfolded protein response (UPR) under conditions of stress or increased folding requirements [25]. The ER contains a transmembrane protein encoded by *IRE1* which has a cytosolic ribonuclease domain and a luminal sensor domain. The sensor detects rising levels of unfolded proteins inside the ER and activates the cytosolic domain. This activity removes an intron from *HAC1* pre-mRNA and the fragments formed are ligated by Trl1p ligase to form mature *HAC1* mRNA. Hac1p is a potent transcriptional activator of UPR target genes which include both *PDI1* and *ERO1*, and dozens of genes related to translocation, glycosylation, folding and degradation [26]. Thus, the UPR balances the biosynthetic rate of secretory proteins and fine tunes the ER capacity to meet cellular demands.

Previous transcription analyses of recombinant xylose-fermenting strains have mainly focused on the changes that occur in genes encoding enzymes involved in central carbon metabolism [27-30]. Very little attention has been given to other fundamental cellular processes such as the folding of proteins. Recently we conducted a comprehensive analysis of the dynamic changes in intracellular metabolites that occur during batch fermentation of a glucose/xylose mixture using a recombinant XR/XDH-strain [31]. In that study we observed a marked accumulation of intracellular malate and fumarate during xylose fermentation. The direct connection between fumarate metabolism and protein folding under anaerobic conditions prompted us to further investigate this system during xylose fermentation. Due to the essential role of FR enzymes in anaerobic growth we hypothesized that the activity of FR is reduced on xylose which in turn would affect the capacity to regenerate the FAD required for proper folding of proteins in the ER. Such an impairment of the protein folding process could be the underlying cause for the slow anaerobic growth on xylose. In this study we examined this hypothesis by over-expressing the two genes encoding FR, alone and in combination with *ERO1*, and evaluated the effect on fermentation performance in two different media containing glucose and xylose.

## Methods

### Strains and culture conditions

Plasmids and yeast strains used in the study are listed in Table 1. Yeast strains were recovered from 20% glycerol stocks stored at −80°C on solid YPD plates (10 g/L yeast extract, 20 g/L peptone, 20 g/L glucose, 15 g/L agar) for two days at 30°C. Yeast cultures were grown in liquid YPD medium for 14–16 h, or less when required, at 30°C and 180 rpm in an orbital shaker. Transformants were selected on solid Yeast Nitrogen Base (YNB) medium (6.7 g/L YNB with ammonium sulphate and without amino acids) with 20 g/L glucose as carbon source. Leucine was supplemented when required at 220 mg/L. Cultivations in shake flasks had a culture volume of 10% or less of the flask volume. The defined medium used in fermentation experiments contained 2X YNB, 20 g/L glucose, 50 g/L xylose and 50 mM potassium hydrogen phthalate buffer at pH 5.5. The complex medium used in fermentation experiments contained 10 g/L yeast extract, 20 g/L glucose, 50 g/L xylose, 50 mM potassium hydrogen phthalate buffer at pH 5.5, 10 g/L (NH_4_)_2_SO_4_, 1 g/L MgSO_4_ and 6 g/L KH_2_PO_4_. All media used in anaerobic cultivations were supplemented with Tween 80 (400 mg/L) and ergosterol (10 mg/L).

**Table 1:** Strains and plasmids used in this study

**Name**	**Relevant genotype**	**Reference**
**Plasmids**		
p426TEF	*URA3*	[32]
YIplac128	*LEU2*	[33]
YIplac211	*URA3*	[33]
YIpDR1	YIplac128:*TDH3*p-*GXF1*-*CYC1*t	[34]
YIpDR7	YIplac211:*TDH3*p-*XYL1*(N272D)-*ADH1*t; *PGK1*p-*XYL2*-*PGK1*t	[35]
YIpBB1	YIplac128:*TDH3*p-*FRD1*-*CYC1*t	This study
YIpBB2	YIplac128:*TDH3*p-*OSM1*-*CYC1*t	This study
YIpBB3	YIplac128:*TDH3*p-*FRD1*-*CYC1*t; *TEF1*p-*ERO1*-*ERO1*t	This study
YIpBB4	YIplac128:*TDH3*p-*OSM1*-*CYC1*t; *TEF1*p-*ERO1*-*ERO1*t	This study
**Yeast strains**		
TMB3043	CEN.PK2-1C; *gre3*-Δ; *his3*::*PGK1*p-*XKS1*-*PGK1*t, *HIS3*; *tal1*::*PGK1*p-*TAL1*-*PGK1*t; *tkl1*::*PGK1*p-*TKL1*-*PGK1*t; *rki1*::*PGK1*p-*RKI1-PGK1t*; *rpe1*::*PGK1*p-*RPE*1-*PGK1*t; *ura3*, *leu2*	[36]
TMB3452	TMB3043; *ura3*::YIpDR7; *leu2*	This study
TMB3455	TMB3452; *leu2*::YIplac128	This study
TMB3456	TMB3452; *leu2*::YIpBB1	This study
TMB3457	TMB3452; *leu2*::YIpBB2	This study
TMB3458	TMB3452; *leu2*::YIpBB3	This study
TMB3459	TMB3452; *leu2*::YIpBB4	This study

*E. coli* strain NEB5α (New England Biolabs, USA) was used for sub-cloning of plasmid DNA. Transformants were selected on solid LB plates (5 g/L yeast extract, 10 g/L tryptone, 10 g/L NaCl, 15 g/L agar, pH 7.0), supplemented with 100 mg/L of ampicillin, for 16 h at 37°C. Cultures of transformed *E. coli* were recovered from 25% glycerol stocks stored at −80°C and grown in liquid LB medium, supplemented with ampicillin, for 14–16 h at 37°C and 180 rpm in an orbital shaker.

### Cloning procedures

Standard molecular biology techniques were used for all cloning procedures [37]. Plasmid purification was performed using GeneJet Plasmid Mini-prep Kit (Thermo Scientific, USA). DNA fragments digested with restriction enzymes were excised from agarose gel and purified using QIAquick Gel Extraction Kit (Qiagen, Germany) when needed. DNA fragments amplified by PCR were purified using GeneJet PCR Purification Kit (Thermo Scientific, USA). All enzymes for PCR amplifications, ligations and restriction digestions were obtained from Thermo Scientific unless stated otherwise. PCR products were amplified using the High Fidelity Phusion Hotstart II Polymerase or High Fidelity Taq Polymerase Mix (Thermo Scientific, USA). Competent yeast cells were prepared and transformed according to the PEG/LiAc method [38] and heat shock competent *E. coli* cells were prepared according to the Inoue method [37] and transformed according to the supplier’s instructions.

The gene *FRD1* was amplified from yeast genomic DNA by PCR using primers FRD1-F1-SpeI (5′-GAC TAG TAA ATG TCT CTC TCT CCC GTT G-3′) and FRD1-R1-SalI (5′-GTT GTC GAC TTA CTT GCG GTC ATT GGC-3′). The gene *OSM1* was amplified from yeast genomic DNA by PCR using primers OSM1-F1-SpeI (5′-GAC TAG TAA AAT GAT TAG ATC TGT GAG-3′) and OSM1-R1-SalI (5′-GTT GTC GAC TCA GTA CAA TTT TGC TAT G-3′). The amplified *FRD1* and *OSM1* genes were digested with *Spe*I and *Sal*I and inserted between the *TDH3* promoter and *CYC1* terminator of the previously constructed vector YIpDR1, yielding the two plasmids YIpBB1 and YIpBB2 (Table 1). The cloned genes were verified by DNA sequencing.

The *ERO1* gene, including a 300 bp downstream sequence, was amplified from yeast genomic DNA by PCR using primers ERO1-F1-BamHI (5′-GAG GAT CCA AAA TGA GAT TAA GAA CCG CCA TTG-3′) and ERO1-R1-XhoI (5′-GTC TCG AGC CCT TGA AGA TGG TAC C-3′). The amplified *ERO1* ORF and terminator was digested with *Bam*HI and *Xho*I and inserted between the *TEF1* promoter and *CYC1* terminator of the vector p426TEF (Table 1). The *TEF1*p-*ERO1*-*ERO1*t cassette was amplified from plasmid p426TEF-ERO1 using primers p426TEF-F1-SacI (5′-GGA GCT CAT AGC TTC AAA ATG-3′) and p426ERO1t-R1-NdeI (5′-CAT ATG CCC TTG AAG ATG GTA C-3′). The amplified cassette was digested with *Bam*HI and *Xho*I and ligated into plasmids YIpBB1 and YIpBB2, digested with the same enzymes, yielding vectors YIpBB3 and YIpBB4, respectively (Table 1). The cloned cassette was verified by DNA sequencing.

### Strain construction

*S. cerevisiae* strain TMB3452 was constructed by transforming strain TMB3043 with YIpDR7, linearized with *Eco*RV. The integrative plasmids YIpBB1-4 were linearized with *Eco*RV, *Afl*II, *Eco91*I and *Afl*II, respectively, and used to transform strain TMB 3452, yielding strains TMB3456 (*FRD1*), TMB3457 (*OSM1*), TMB3458 (*FRD1 ERO1*) and TMB3459 (*OSM1 ERO1*) (Table 1). The control strain TMB3455 was constructed by transforming TMB3452 with YIplac128, linearized with *Eco*RV. Correct integration of the vectors was verified by PCR using primer pairs that annealed on the plasmid and on the chromosome outside the homologous regions of *URA3* or *LEU2* loci. The functionality of the over-expressed *FRD1* and *OSM1* genes was verified by measuring the specific fumarate reductase activity in cell-free protein extracts from strains TMB3455-59 grown anaerobically on xylose. Strains TMB3456-59 had between 9- and 30-fold higher activities than the control showing that the over-expressed genes generated increased levels of functional enzymes (Additional file 1: Figure S1). The functionality of the over-expressed *ERO1* gene was verified by determining the sensitivity toward DTT. Single colonies of strains TMB3455-59 were inoculated in 5 mL YNB medium with 2% glucose and grown over-night. 200 μL of this culture was used to inoculate fresh medium and cultivated until the cells reached OD_620 nm_ ≈ 1. A volume containing 2 × 10^6^ cells (150–200 μL) was spread on solid YPD medium after which a filter disc was placed in the centre of the plate and soaked with 20 μL or 30 μL of 1 M DTT solution. The plates were incubated 48 h at 30°C before measuring the diameter of the halo formed around the disc. The halos formed with strains TMB3458 and TMB3459 were 20-30% smaller compared with the other strains showing that the over-expressed *ERO1* gene generated increased level of a functional enzyme (Additional file 1: Figure S2).

### Anaerobic batch fermentations

Yeast strains were pre-grown in YNB medium with 2% glucose until mid-exponential phase and inoculated into 50 mL medium with 2% glucose and 5% xylose in 250 mL crimp-sealed serum flasks at a concentration of 0.04 g CDW/L. Prior to inoculation the headspace was flushed with N_2_-gas containing less than 5 ppm oxygen (AGA Gas AB, Sweden) for at least 30 min to ensure anaerobic conditions. Cultivations were carried out in a 30°C water bath with continuous stirring at 180 rpm using magnetic stirrers. To prevent diffusion of oxygen into the culture during sampling, the excess gas was released through a 0.2 μm filter connected to Norprene tubing submerged under water. Cell dry weight was calculated from OD measurements using a pre-determined relationship between OD and cell dry weight. Fermentation experiments were performed in biological duplicates.

### Preparation of cell-free protein extracts

Yeast strains were cultivated anaerobically in crimp-sealed serum flasks as described above using 2X YNB medium with 2% glucose and 5% xylose. After 36 h, when xylose was the only carbon source, the cells were harvested by centrifugation at 4000 rpm and 4°C for 10 min. The resulting cell pellets were resuspended in 1X Lysis buffer (20 mM K,Na-PO_4_, 20 mM EDTA, 1 mM DTT, pH 7.0) to obtain a final suspension of 1 g wet cells/mL. Protein extracts were prepared using a Precellys®24 homogenizer with cooling provided by the Cryolys® unit (Bertin Technologies, France) and Precellys Lysing Kit VK05 containing 0.5 mm glass beads. The unit was operated for 3 × 45 s at 5000 rpm with 30 s pauses between each cycle and at max 8°C. Cell debris was pelleted by centrifugation at 14,000 × *g* and 4°C for 15 min and the supernatant was transferred to a small glass vial. Before sealing the vial the headspace was replaced with N_2_-gas in an anaerobic chamber. The protein extracts were stored at 4°C until the following day. Protein concentrations were determined with the Coomassie Protein Assay Reagent (Thermo Scientific, USA) using BSA as standard (Thermo Scientific, USA).

### Fumarate reductase activity assay

Fumarate reductase activity was determined in a 1.2 mL reaction mixture containing: 50 mM K,Na-PO_4_ buffer (pH 7.3), 10 mM fumaric acid (pH 7.3), 0.4 mM FMN-Na, 2% (w/v) sodium hydrosulfite. All reagents, except protein extract, were added to a rubber-sealed cuvette inside an anaerobic chamber. The assay buffer was prepared by dissolving 2.762 g of KH_2_PO_4_ and 2.837 g of Na_2_HPO_4_·2H_2_O in 50 mL anaerobic water (separately) inside the anaerobic chamber, creating one 10X solution and one 2X solution, respectively. A 200 mM stock solution at the correct pH was made by mixing 5 mL 10X with 25 mL 2X and completing the volume to 50 mL with anaerobic water. A 40 mM stock solution of fumaric acid was prepared by dissolving 0.464 g fumaric acid in 50 mL of 5 mM K,Na-PO_4_ buffer at pH 7.3. The pH was adjusted to 7.3 using 5 M NaOH and the volume was completed to 100 mL with water. Solutions of the cofactor and reducing agent were prepared fresh before each assay. Both compounds were weighed and brought into the anaerobic chamber. The FMN cofactor was dissolved in anaerobic water and sodium hydrosulfite was dissolved in 100 mM K,Na-PO_4_ buffer, but only after all other reagents had been added to the cuvette to ensure that FMN becomes completely reduced to FMNH_2_. The reaction was started by addition of protein extract using a gas-tight syringe. The formation of FMN was measured at 445 nm in cuvettes with light path length of 1 cm using an Ultrospec 2100 Pro spectrophotometer (Amersham Biosciences Corp., USA) controlled by computer software SWIFT Reaction Kinetics v. 2.05 (Biochrom Ltd., Cambridge, UK). The absorbance was converted to concentration units using the molar extinction coefficient of FMN (ϵ_445 nm_ = 12,500 L/mol/cm). The slope was determined within an interval of at least 1 min, using at least three different dilutions of the protein extract. Only the slopes within the range 0.045-0.40 A/min were considered acceptable. One unit is defined as the conversion of 1 μmol of fumarate per minute.

### Biomass determination and analysis of metabolites

Optical density was measured at 620 nm using an Ultrospec 2100 Pro spectrophotometer (Amersham Biosciences Corp., USA). Cell dry weight was measured in triplicate by filtering a known volume of the culture through a pre-weighed nitrocellulose filter with 0.45 μm pore size. The filters were washed with three volumes of water, dried in a microwave oven and weighed after equilibrating to room temperature in a desiccator.

Concentrations of glucose, xylose, xylitol, glycerol, succinate, acetate and ethanol were analysed by HPLC (Waters, USA). Aminex HPX-87H ion exchange column (Bio-Rad, USA) was used at 45°C with a mobile phase of 5 mM H_2_SO_4_ at a flow rate of 0.6 mL/min. All compounds were detected with a RID-10A refractive index detector (Shimadzu, Japan).

### Calculation of metabolic rates

In defined medium (YNB) the volumetric production and consumption rates (*q*_*i*_ and *q*_*s*_), the maximum specific consumption rates of xylose (*r*_*xyl*_) and glucose (*r*_*glc*_), as well as the biomass yield per consumed substrate (*Y*_*x/s*_) were calculated through linear regression according to Equations 1–4. The product yields per consumed substrate (*Y*_*i/s*_) were calculated using the determined volumetric rates according to Equation 5. The product yields per biomass (*Y*_*i/x*_) were calculated as the ratio between *Y*_*i/s*_ and *Y*_*x/s*_ when xylose was the carbon source and according to Equation 6 when glucose was the carbon source. Due to the non-exponential growth on xylose, the maximum specific growth rate (*μ*_max_) was calculated as the product between *r*_*xyl*_ and *Y*_*x/s*_ according to Equation 7. For comparative reasons *μ*_max_ on glucose was calculated in the same way. These values corresponded well with those calculated through regression of ln(OD) vs. time. The specific production rates were calculated as the product between *μ*_max_ and *Y*_*i/x*_ according to Equation 8.

(1) qi=dCidt,qs=dCsdt

(2) $$r_{\mathrm{xyl}}=\frac{d\left(C_{\mathrm{xyl}}/C_{x}\right)}{\mathrm{dt}}$$

(3) $$r_{\mathrm{glc}}=\frac{d\left(dC_{\mathrm{glc}}/\mathrm{dt}\right)}{dC_{x}}$$

(4) $$Y_{x/s}=\frac{dC_{x}}{dC_{s,\mathrm{tot}}}$$

(5) $$Y_{i/s}=\frac{q_{i}}{q_{s}}$$

(6) $$Y_{i/x}=\frac{dC_{i}}{dC_{x}}$$

(7) $$\mu_{\text{max}}=r_{s}\cdot Y_{x/s}$$

(8) $$r_{i}=\mu_{\text{max}}\cdot Y_{i/x}$$

In complex medium all calculations were made as described above except for the maximum specific consumption rate of glucose which could not be determined accurately enough. Hence, the *μ*_max_ under these conditions was determined as the slope in a linear regression of ln(OD) vs. time and *r*_*glc*_ was calculated using Equation 7. The significance of a difference between two mean values was calculated using a two-tailed t-test assuming equal variance of the two samples with two degrees of freedom (*n*_1_ = *n*_2_ = 2) unless stated otherwise. Balances of carbon and degree of reduction, determined for the glucose and xylose phases using calculated yield coefficients, are given as supplementary information in Additional file 2: Table S1.

### Transcriptional analysis of UPR related genes

The list of genes reported as targets of the UPR were taken from [26]. Transcript data from cultivations of a XR/XDH-strain using glucose or xylose in aerobic and anaerobic conditions was taken from [27]. UPR target genes that had a significant change in expression (*P* ≤ 0.1) during the transition from aerobic to anaerobic conditions with xylose as carbon source were included in the analysis. Two genes commonly used as targets of the UPR (*KAR2* and *INO1*) [27] did not pass the statistical requirement and were thus not included in the analysis (Additional file 2: Table S5). Hierarchical clustering was performed using the *clustergram* function in MATLAB R2010b with log_2_ values of transcriptional changes. Euclidean distance was used to calculate the distance between the data points and average linkage was used to generate the dendrogram. Data used for the hierarchical clustering is given as supplementary information in Additional file 2: Table S2.

## Results and discussion

### Genes targeted by the unfolded protein response are differentially expressed on xylose compared to glucose

**Figure 2:**
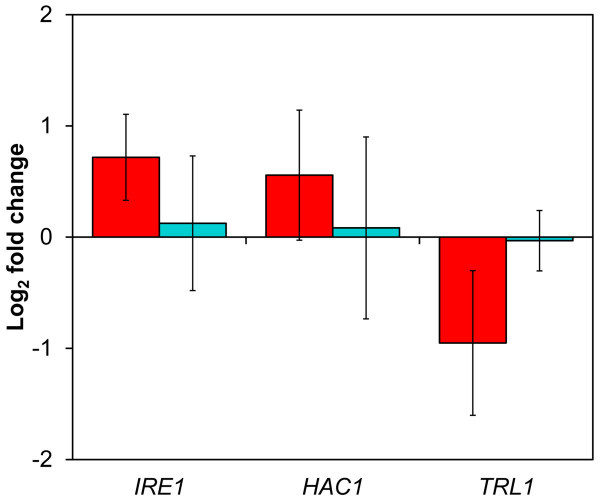
**Transcriptional response of genes involved in the regulation of the UPR.** Bars show the log_2_ fold change of the indicated genes during a transition from aerobic to anaerobic conditions in a XR/XDH-strain utilizing xylose (red bars) or glucose (turquoise bars) as carbon source [27]. Values are given as mean fold change of two independent measurements in each conditions and errors represent the 95% confidence interval of the mean (*df* = 2).

**Figure 3:**
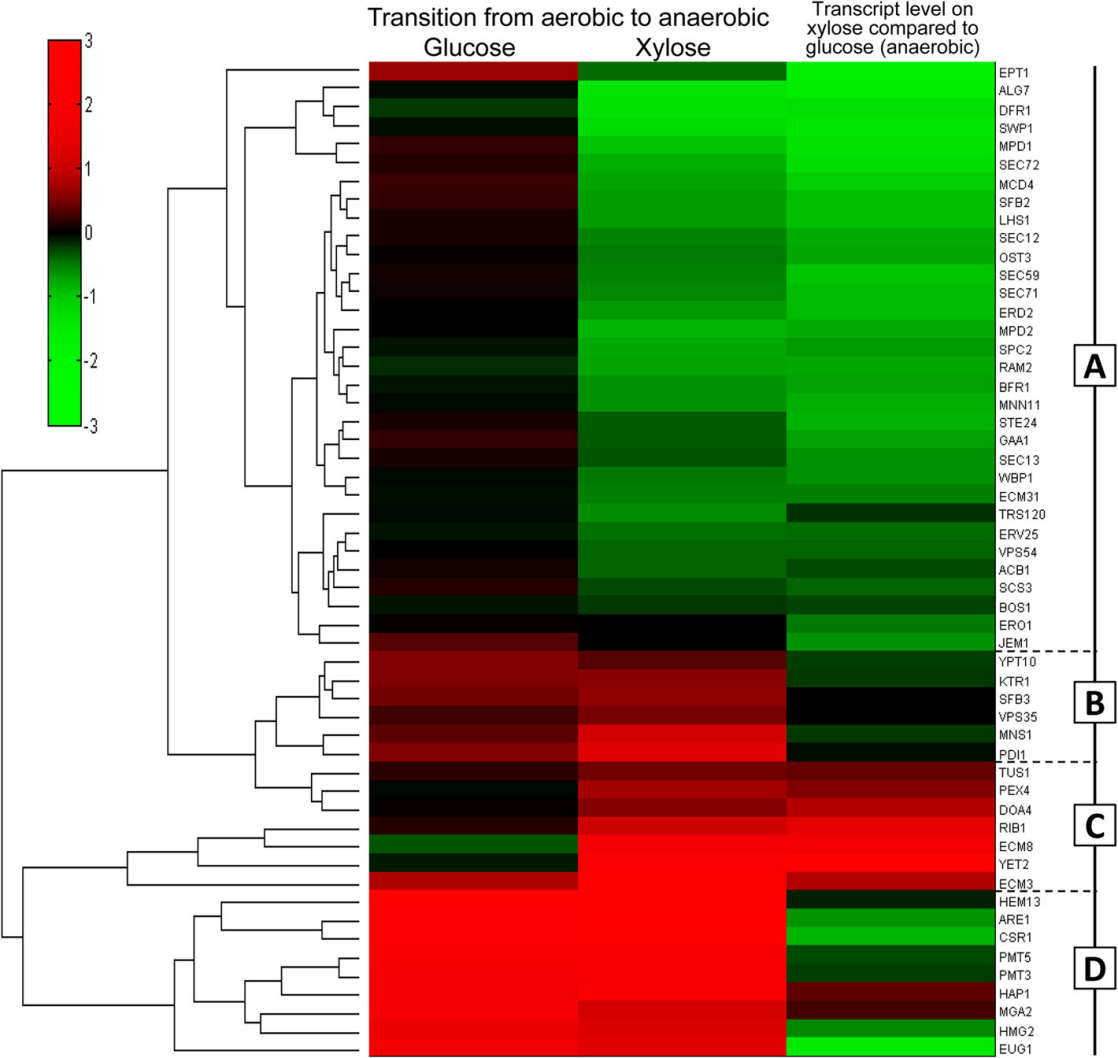
**Hierarchical clustering of genes induced by the UPR.** Genes that are induced by the UPR [26] and changed significantly (*P* ≤ 0.1) during a transition from aerobic to anaerobic conditions in a XR/XDH-strain utilizing xylose [27] were analysed by hierarchical clustering. The heat map was generated using the log_2_ fold change between the different conditions. The analysis shows that a majority (61%) of the included UPR target genes are down-regulated on xylose when the conditions change from aerobic to anaerobic (Group A). These genes do not change during the same transition on glucose indicating that the UPR responds differently to xylose compared to glucose.

A previously performed transcripome study in a recombinant XR/XDH-strain [27] was used to investigate the expression level of genes associated with the UPR. It revealed that *IRE1* and *HAC1* levels were ~65% and ~50% higher (*P* = 0.02 and *P* = 0.05, respectively) in cells growing anaerobically on xylose compared to cells growing aerobically (Figure 2). However, the transcript level of *TRL1*, encoding the ligase, was ~50% lower (*P* = 0.02) (Figure 2), suggesting that *HAC1* is mainly present as fragments rather than mature mRNA molecules. The transcript levels of these genes did not change significantly during the same transition (i.e. from aerobic to anaerobic conditions) on glucose (Figure 2) indicating that the response was associated with the type of carbon source available. The cluster analysis illustrated in Figure 3 indeed shows that a majority (61%) of the included UPR target genes were down-regulated on xylose when the conditions changed from aerobic to anaerobic (Group A). The genes belonging to this group represent functions taking place in all steps of the maturation process (translocation, glycosylation, disulphide bond formation, protein folding, vesicle budding and vesicle transport to the cell membrane) (Additional file 2: Table S2) and do not change during the same transition on glucose. The smallest group (Group B) contained those genes which are regulated equally on glucose and xylose, with only small differences in abundance between the two carbon sources. A small number of genes were up-regulated on xylose while remaining largely unchanged on glucose (Group C). These genes are related to peroxisome biogenesis (*PEX4*), ubiquitin recycling (*DOA4*) and the cell integrity signalling pathway (*TUS1*). The three genes *ECM3*, *ECM8* and *YET2* encode proteins with unknown functions. *RIB1* encodes GTP cyclohydrolase II which catalyses the first step of the riboflavin (FAD and FMN) biosynthesis pathway. The fourth group contains genes which are strongly up-regulated in response to anaerobiosis in both conditions (Group D). However, in contrast to the genes in Group B, the transcript levels of these genes were in general lower on xylose compared to glucose. This analysis shows that the UPR responded differently to xylose compared to glucose. Furthermore, the repression of a majority of the target genes suggests that the capacity of the secretory pathway could be limited during xylose fermentation.

Consistent with previously reported expression patterns on non-fermentable carbon sources and entry into stationary phase [39,40], the expression of *ERO1* was reduced by ~30% (*P* < 0.02) on xylose compared to glucose, both under anaerobic and aerobic conditions (Additional file 2: Table S5) [27]. Hence, a reduced capacity to form disulphide bonds in nascent polypeptides translocated to the ER could be one underlying cause for the low anaerobic growth rate on xylose. The previously reported accumulation of intracellular fumarate during xylose fermentation [31] suggested that recombinant XR/XDH strains have a limited FR activity under such conditions, which would lead to insufficient recycling of the FAD co-factor required by Ero1p. This hypothesis was investigated by evaluating the physiological effect of over-expressing the cytosolic and mitochondrial FR enzymes, alone and in combination with *ERO1*, during fermentation of a mixture of glucose and xylose.

### Over-expression of cytosolic FR has limited impact on xylose fermentation

**Figure 4:**
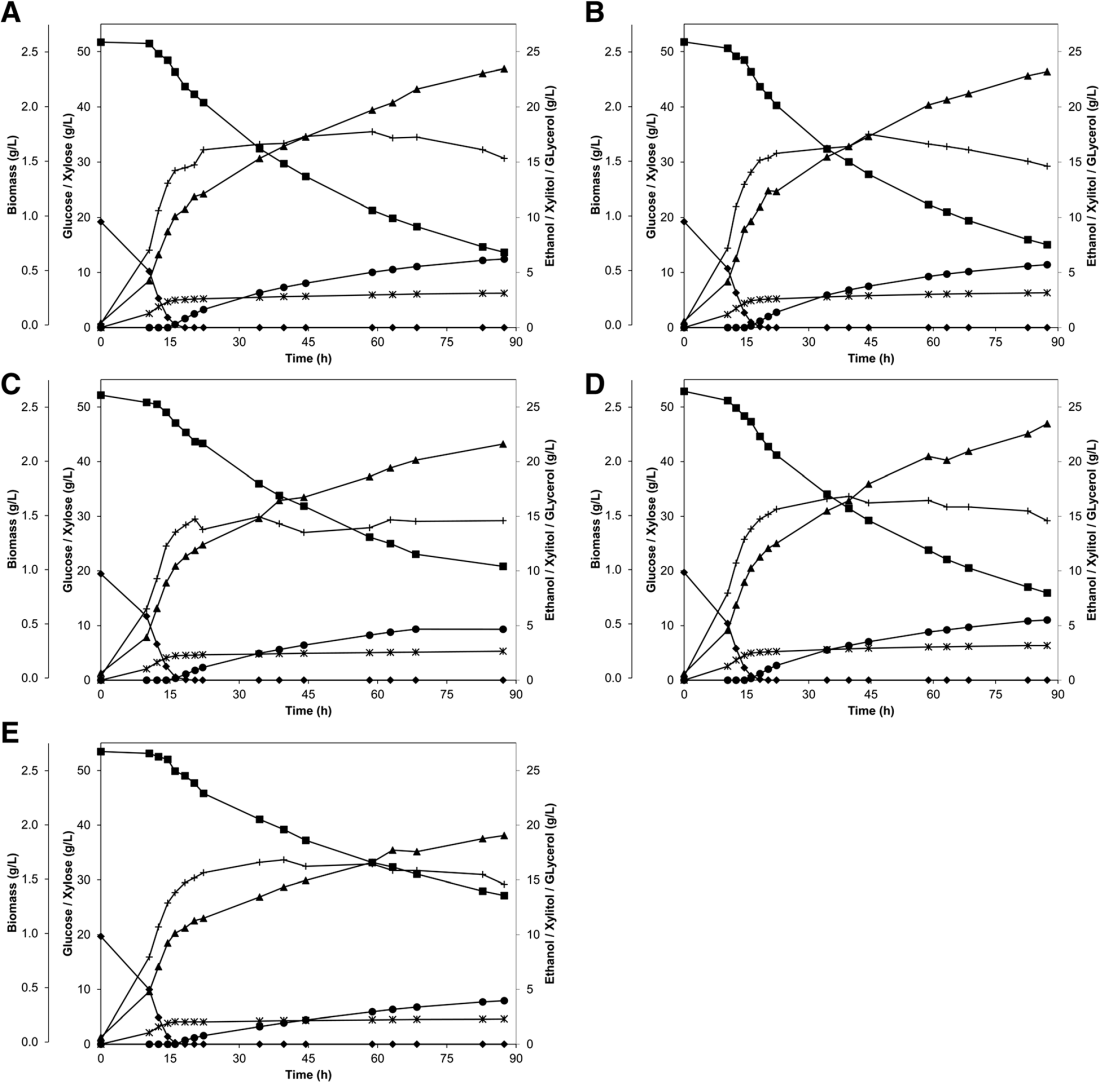
**Fermentation of a glucose/xylose mix in defined medium.** A mix of 20 g/L glucose and 50 g/L xylose was fermented in defined medium (2X YNB) by strains **A)** TMB3455 (control), **B)** TMB3456 (*FRD1*), **C)** TMB3457 (*OSM1*), **D)** TMB3458 (*FRD1 ERO1*) and **E)** TMB3459 (*OSM1 ERO1*). Symbols: diamonds, glucose; squares, xylose; plus, biomass; triangles, ethanol; circles, xylitol; stars, glycerol. Figures illustrate one representative experiment out of two biological replicates.

Due to the higher contribution of the cytosolic FR to the total cellular reductase activity the over-expression of *FRD1,* alone and in combination with *ERO1*, was evaluated first. When cultivated in defined medium (2X YNB) with 20 g/L glucose and 50 g/L xylose, TMB3456 (*FRD1*) and TMB3458 (*FRD1 ERO1*) consumed the glucose within 20 h and ca. 70% of the xylose within 90 h (Figure 4B and D). The two strains behaved very similar to the control strain (Figure 4A) and no significant physiological differences from this strain were observed (Additional file 2: Table S3). These results suggest that the implemented genetic modifications had no positive effect on the anaerobic growth on xylose in defined medium. However, the modifications only ensured that the genes required for regeneration of FAD co-factors were present (Figure 1). If substrates are not available in sufficient amounts (in this case unfolded polypeptides) these modifications are not effective in regenerating the co-factor. The poor repressive capability of xylose leads to an up-regulation of *MDH2*[27] (Additional file 2: Table S5), encoding cytosolic malate dehydrogenase, which has previously been suggested as an unbeneficial reaction that directs carbon away from amino acid synthesis by converting oxaloacetate into malate [31]. A reduced synthesis of amino acids could thus hamper the formation of polypeptides and mask the effect of the genetic modifications. To investigate whether the strains were limited by amino acid synthesis they were cultivated in a complex medium containing yeast extract. Changing from a defined medium to a complex medium led to several significant changes in the physiology of all strains, e.g. increased maximum growth rate, increased biomass yield and reduced by-product yields (Additional file 2: Table S3 and Table S4). Hence, the glucose was consumed within 15 h and ca. 80% of the xylose was consumed within 65 h (Additional file 1: Figure S3). However, despite the increased nutrient availability no significant differences were observed between the control strain and the *FRD1*-overexpressing strains TMB3456 and TMB3458 (Additional file 2: Table S4).

### Over-expression of mitochondrial FR has a detrimental effect on xylose fermentation

Although the change from a defined medium to a complex medium led to a general increase in the growth rate on xylose, the over-expression of *FRD1*, alone or in combination with *ERO1*, had little effect on the cellular physiology. One explanation could be that the previously observed accumulation of fumarate [31] does not occur in the cytosol, but is restricted to the mitochondria. To investigate this possibility we over-expressed the *OSM1* gene, alone and in combination with *ERO1*, and evaluated the fermentation performance in both defined and complex media with 20 g/L glucose and 50 g/L xylose.

In defined medium, TMB3457 (*OSM1*) and TMB3459 (*OSM1 ERO1*) also consumed the glucose within 20 h and ca. 60% of the xylose within 90 h (Figure 4C and E). During glucose fermentation these strains behaved similar to the other strains and gave nearly the same product yields as the control strain (Table 2). When xylose became the sole carbon source the physiology changed drastically and the two strains behaved differently. The maximum growth rate of strain TMB3457 (*OSM1*) was 47% lower (*P* = 0.03) compared with the control strain (Table 2) and the biomass-specific yields of acetate and ethanol increased by 130% (*P* = 0.04) and 69% (*P* = 0.05), respectively (Table 2). As a result a smaller fraction of the carbon was distributed to biomass and glycerol in favour of acetate and ethanol (Table 2). Strain TMB3459 (*OSM1 ERO1*) did not display any difference in growth rate on xylose compared with the control strain (Table 2). The acetate yield per biomass also increased by 44% (*P* = 0.008) whereas the glycerol and xylitol yields decreased by 39% (*P* = 0.08) and 20% (*P* = 0.002) compared with the control strain (Table 2). Hence, this strain diverted the carbon away from glycerol, xylitol and ethanol for a higher yield of acetate and maintained biomass yield (Table 2).

**Table 2:** Maximum specific growth rates and product yields in anaerobic batch fermentation of 20 g/L glucose and 50 g/L xylose in defined medium (2X YNB) using strains TMB3455, TMB3457 and TMB3459

	**Glucose phase**			**Xylose phase**		
	**TMB3455 (control)**	**TMB3457 (*OSM1*)**	**TMB3459 (*OSM1 ERO1*)**	**TMB3455 (control)**	**TMB3457 (*OSM1*)**	**TMB3459 (*OSM1 ERO1*)**
*μ*_*max*_ (1/h)	0.29 ± 0.012	0.30 ± 0.019	0.32 ± 0.044	0.029 ± 0.002	0.018 ± 0.001	0.028 ± 0.003
**Yields per substrate (mol/mol sugar)**						
*Y*_*xylt/s*_	–	–	–	0.24 ± 0.03	0.25 ± 0.01	0.20 ± 0.02
*Y*_*glyc/s*_	0.22 ± 0.02	0.20 ± 0.02	0.20 ± 0.05	0.020 ± 0.000	0.016 ± 0.002	0.012 ± 0.001
*Y*_*ac/s*_	0.020 ± 0.001	0.012 ± 0.000	0.017 ± 0.005	0.019 ± 0.001	0.031 ± 0.003	0.029 ± 0.003
*Y*_*etoh/s*_	1.87 ± 0.14	1.96 ± 0.21	1.96 ± 0.37	1.03 ± 0.00	1.24 ± 0.15	0.87 ± 0.00
*Y*_*succ/s*_	0.003 ± 0.000	0.001 ± 0.000	0.002 ± 0.001	0.009 ± 0.000	0.007 ± 0.002	0.006 ± 0.000
*Y*_*x/s*_ (g/mol)	10.4 ± 0.5	10.5 ± 0.2	11.9 ± 0.9	3.8 ± 0.4	2.7 ± 0.6	3.9 ± 0.4
**Yields per biomass (mmol/g CDW)**						
*Y*_*xylt/x*_	–	–	–	63.1 ± 0.3	92.9 ± 25.8	50.3 ± 0.8
*Y*_*glyc/x*_	20.2 ± 0.8	18.3 ± 0.5	16.6 ± 0.2	5.2 ± 0.6	5.8 ± 0.7	3.2 ± 0.6
*Y*_*ac/x*_	2.1 ± 0.1	1.8 ± 0.1	1.8 ± 0.0	5.1 ± 0.3	11.7 ± 1.8	7.3 ± 0.0
*Y*_*etoh/x*_	153 ± 8	153 ± 11	147 ± 7	271 ± 30	458 ± 53	223 ± 23
*Y*_*succ/x*_	0.19 ± 0.04	0.28 ± 0.18	0.22 ± 0.04	2.5 ± 0.4	2.4 ± 0.3	1.6 ± 0.22

Values are given as mean ± standard deviation of two independent experiments. Data for strains TMB3456 and TMB3458 is reported in Additional file 2: Table S3.

In complex medium strains TMB3457 (*OSM1*) and TMB3459 (*OSM1 ERO1*) consumed glucose within 15 h but only 50-60% of xylose was consumed within 65 h (Additional file 1: Figure S3). The performance of the strains did not differ significantly from the control strain during the glucose phase (Table 3). With xylose as the carbon source, on the other hand, both strains grew markedly slower than the control strain. The growth rates of TMB3457 (*OSM1*) and TMB3459 (*OSM1 ERO1*) on xylose were reduced by 25% (*P* = 0.09) and 51% (*P* = 0.12), respectively (Table 3). Both strains also increased the biomass-specific yield of acetate by 73% (*P* = 0.003) and 157% (*P* = 0.03), respectively (Table 3). Although both strains diverted more carbon toward acetate, different by-product yields were reduced by each strain. TMB3457 (*OSM1*) gave a reduced substrate-specific yield of glycerol by 31% (*P* = 0.09) whereas TMB3459 (*OSM1 ERO1*) gave reduced yields of ethanol and biomass by 21% (*P* = 0.06) and 34% (*P* = 0.11), respectively (Table 3).

**Table 3:** Maximum specific growth rates and product yields in anaerobic batch fermentation of 20 g/L glucose and 50 g/L xylose in complex medium (10 g/L yeast extract) using strains TMB3455, TMB3457 and TMB3459

	**Glucose phase**			**Xylose phase**		
	**TMB3455 (control)**	**TMB3457 (*OSM1*)**	**TMB3459 (*OSM1 ERO1*)**	**TMB3455 (control)**	**TMB3457 (*OSM1*)**	**TMB3459 (*OSM1 ERO1*)**
*μ*_*max*_ (1/h)	0.37 ± 0.003	0.38 ± 0.001	0.38 ± 0.003	0.033 ± 0.004	0.024 ± 0.000	0.016 ± 0.008
**Yields per substrate (mol/mol sugar)**						
*Y*_*xylt/s*_	–	–	–	0.14 ± 0.01	0.16 ± 0.01	0.13 ± 0.01
*Y*_*glyc/s*_	0.13 ± 0.01	0.11 ± 0.01	0.11 ± 0.01	0.013 ± 0.001	0.009 ± 0.001	0.009 ± 0.002
*Y*_*ac/s*_	0.030 ± 0.006	0.022 ± 0.000	0.025 ± 0.003	0.022 ± 0.003	0.037 ± 0.005	0.037 ± 0.001
*Y*_*etoh/s*_	2.7 ± 0.6	2.6 ± 0.2	2.6 ± 0.2	1.4 ± 0.1	1.4 ± 0.0	1.1 ± 0.0
*Y*_*succ/s*_	0.005 ± 0.004	0.003 ± 0.001	0.003 ± 0.001	0.003 ± 0.001	0.006 ± 0.000	0.005 ± 0.001
*Y*_*x/s*_ (g/mol)	17.5 ± 1.4	18.4 ± 0.9	19.5 ± 0.6	6.4 ± 0.8	6.3 ± 0.7	4.2 ± 0.8
**Yields per biomass (mmol/g CDW)**						
*Y*_*xylt/x*_	–	–	–	22.3 ± 0.8	25.5 ± 1.2	31.0 ± 8.7
*Y*_*glyc/x*_	7.3 ± 0.5	6.4 ± 0.2	6.4 ± 0.0	2.1 ± 0.0	1.5 ± 0.4	2.3 ± 0.9
*Y*_*ac/x*_	1.3 ± 0.4	1.3 ± 0.1	1.5 ± 0.1	3.4 ± 0.0	5.9 ± 0.2	8.8 ± 1.4
*Y*_*etoh/x*_	94.2 ± 5.0	91.3 ± 5.9	93.0 ± 3.5	222 ± 11	228 ± 21	272 ± 62
*Y*_*succcxxx*_	0.12 ± 0.07	0.17 ± 0.08	0.21 ± 0.02	0.54 ± 0.03	0.91 ± 0.03	1.2 ± 0.5

Values are given as mean ± standard deviation of two independent experiments. Data for strains TMB3456 and TMB3458 is reported in Additional file 2: Table S4.

These results indicate that the reactions taking place in the mitochondria have a significant role in xylose metabolism and that they are more sensitive to changes in metabolite levels (leading to altered regulatory signals, degree of allosteric inhibition of enzymes etc.) on xylose compared to glucose. Hence, the over-expression of *OSM1* caused physiological responses that were not observed in strains over-expressing *FRD1*. In strain TMB3457 (*OSM1*) these responses were: i) a 20-40% reduction in glycerol yield, ii) a 63-68% increase in acetate yield and iii) a 27-38% reduction in growth rate during xylose fermentation (Table 2 and Table 3). A possible explanation for this phenotype could be that Osm1p interacts with Gut2p and Ach1p which are both located specifically inside the mitochondria (Additional file 1: Figure S4). *GUT2* encodes a FAD-dependent glycerol 3-phosphate dehydrogenase located in the mitochondrial inner membrane [41] and expression of the gene is repressed in the presence of glucose and induced during growth on non-fermentable carbon sources [42]. An induction of the *GUT2* gene has also been shown to occur during xylose fermentation with transcript levels more than 7-fold higher compared to glucose [27] (Additional file 2: Table S5). Recently, the protein encoded by *ACH1* was shown to function as a Coenzyme A (CoA) transferase rather than an acetyl-CoA hydrolase [43]. Hence, this enzyme catalyses the following reaction: acetyl-CoA + succinate → succinyl-CoA + acetate (Δ_r_G’° = −8.1 kJ/mol) and is also induced nearly 5-fold during xylose fermentation compared to glucose [27] (Additional file 2: Table S5). If both Gut2p and Ach1p are active on xylose, the pathway formed together with Osm1p could explain the diversion of carbon from glycerol to acetate as well as the reduced growth rate due to a reduced acetyl-CoA availability (Additional file 1: Figure S4). However, this hypothesis needs to be validated through further investigations.

### Frd1p and Osm1p do not influence regeneration of cytosolic NAD

An early study on the role of FR showed that the addition of oxidised methylene blue or phenazine methosulfate, which chemically oxidises NADH to NAD, to cultures of a *frd1*Δ *osm1*Δ double mutant rescued the non-growing phenotype under anaerobic conditions [17]. It was thus proposed that the two FR enzymes are involved in the oxidation of excess NADH formed in the cytosol and the mitochondria under anaerobic conditions. The formation of xylitol during xylose fermentation has been ascribed to an inability of the cells to regenerate the NAD needed for the conversion of xylitol to xylulose by XDH [44]. The addition of acetoin, a substrate which can be reduced by yeast to 2,3-butanediol using NADH, to cultures fermenting xylose reduces the xylitol yield significantly [45,46]. If the FR enzymes participate in the conversion of excess NADH a reduction of the xylitol yield during xylose fermentation would be expected when the FR activity is increased. However, the over-expression of *FRD1* or *OSM1* did not affect the amount of xylitol produced from xylose (Table 2, Table 3, Additional file 2: Table S3 and Table S4). This is in agreement with other studies showing that addition of acetoin to a culture of a *frd1*Δ *osm1*Δ double mutant does not rescue the non-growing phenotype under anaerobic conditions [11]. In addition, the proposed mechanism of NADH oxidation requires the presence of an enzyme capable of transferring electrons from NADH to FAD to generate the reduced cofactor used as substrate by fumarate reductase [11,17]. As of yet, no such enzyme has been identified in *S. cerevisiae* that is expressed under anaerobic conditions. There are, however, FR enzymes that use NADH as co-factor instead of FADH_2_. The NADH-dependent FR enzyme from *Trypanosoma brucei* has recently been expressed in a xylose-utilizing *S. cerevisiae* strain which resulted in increased ethanol yield and decreased xylitol yield when fermenting xylose [47]. Based on these results we conclude that the FR enzymes from *S. cerevisiae* do not influence the regeneration of cytosolic NAD.

### Other potential limitations in the secretory pathway in *S. cerevisiae* during xylose fermentation

The results obtained in this study indicate that the activities of FR and Ero1p enzymes are not limiting during xylose fermentation. Measurements of the FR activity in strain TMB3455 when growing on glucose and when metabolizing xylose indeed showed equal activities around 3 mU/mg protein in both conditions (Additional file 1: Figure S5). This is contradicting previous studies showing that the activity and expression of *FRD1* and *OSM1* decrease upon entry into stationary phase [11,39] and during carbon starvation [48]. It is, however, possible that the FR activity is maintained high on xylose in response to cellular stress. The up-stream regions of the *FRD1* and *OSM1* genes contain binding sites for stress-related transcription factors Yap1p and, in the case of *FRD1*, Msn2p/Msn4p [49]. Due to the involvement of Yap1p, an oxidative stress would be most likely in this case [50]. This possibility is supported by much higher intracellular concentrations of both reduced and oxidized glutathione [31] and higher transcript levels of *MSN4* and *YAP1*[27] in recombinant *S. cerevisiae* strains during xylose fermentation compared to glucose fermentation (Additional file 2: Table S5).

The up-regulation of *RIB1* (Figure 3) could be an indication that the need for flavin-containing complexes and/or the free co-factor is increased on xylose. Ero1p has been shown to use free FAD as electron acceptor *in vitro* under anaerobic conditions [51] which is in line with the ability of the FR enzymes to use free FADH_2_ as cofactor and their essential nature under such conditions. As *S. cerevisiae* possesses one gene encoding a mitochondrial FAD transporter (*FLX1*) [52] and three genes encoding putative transporters associated with the ER (*FLC1-3*) [53], FAD-exchange between compartments should indeed be possible. This highlights free FAD as a potentially crucial metabolite for sustaining anaerobic growth.

Pdi1p is, together with Ero1p, essential for disulphide bond formation in the ER [54]. Although transcriptional analysis did not show any difference in expression of *PDI1* between glucose and xylose fermentation [27] (Additional file 2: Table S5), proteome analysis of a mutant strain with improved xylose-fermenting capability identified Pdi1p as significantly more abundant in the improved strain compared with the wild-type [55]. This strongly indicates that there indeed is a need for a higher capacity in the protein folding mechanism for efficient xylose fermentation. Whether this capacity is for regulation of disulphide bond formation [56], reduction of non-native disulphide bonds [57] or targeting unfolded glycoproteins for degradation [58] remains to be investigated.

## Conclusions

Over-expression of *FRD1*, encoding the cytosolic FR, did not improve the growth rate on xylose, even when the strain was provided with all amino acids and an additional copy of the *ERO1* gene controlled by a strong promoter. Over-expression of the *OSM1* gene, encoding the mitochondrial FR, even had a detrimental effect and caused a reduced growth rate. Hence, we conclude that increasing the activities of the FR enzymes and Ero1p is not sufficient to increase the anaerobic growth on xylose. However, our transcription analysis of UPR related genes showed a wide down-regulation of the secretory pathway during xylose fermentation indicating that additional components of the protein folding mechanism may be limited. Two immediate factors should notably be investigated further: i) the actual form of *HAC1* inside the cells during xylose fermentation to determine the regulatory component and ii) the activity of Pdi1p which is acting together with Ero1p in the formation of disulphide bridges. Furthermore, understanding the requirement of free FAD for anaerobic growth could prove to be essential for the development of efficient xylose-utilizing *S. cerevisiae* strains.

## Abbreviations

AC: Acetate; ACALD: Acetaldehyde; ACCOA: Acetyl-CoA; AKG: Alpha-ketoglutarate; CoA: Coenzyme A; CIT: Citrate; ER: Endoplasmic reticulum; ETOH: Ethanol; FR: Fumarate reductase; FUM: Fumarate; ICIT: Isocitrate; MAL: L-malate; OAA: Oxaloacetate; PYR: Pyruvate; SUCC: Succinate; SUCCOA: Succinyl-CoA; UPR: Unfolded protein response; XDH: Xylitol dehydrogenase; XR: Xylose reductase.

## Competing interests

The authors declare that they have no competing interests.

## Authors’ contribution

The study was conceived by BB who designed the experiments and supervised the master student who performed most of the molecular biology work. The enzymatic assay was developed by BB who also performed the fermentation experiments, analysed the data and wrote the manuscript. BB, MGG and EVN have discussed the results and implications throughout the work. MGG and EVN have commented on the manuscript at all stages. All authors read and approved the final manuscript.

## Supplementary Material

Additional file 1 **Supplementary Figures S1-S5.** Description: FR activity measurements, DTT sensitivity results, fermentation profiles. Click here for file

Additional file 2 **Supplementary Tables S1-S5.** Description: Genes included in the cluster analysis, maximum specific growth rates and product yields using strains TMB3455, TMB3456 and TMB3458. Click here for file

## References

[B1] Visser W Scheffers WA der Vegte WH B-v van Dijken JP Oxygen requirements of yeasts Appl Environ Microbiol 1990 56 12 3785 3792 2082825 10.1128/aem.56.12.3785-3792.1990 PMC185068

[B2] Jönsson L Alriksson B Nilvebrant N-O Bioconversion of lignocellulose: inhibitors and detoxification Biotechnol Biofuels 2013 6 1 16 10.1186/1754-6834-6-16 23356676 PMC3574029

[B3] Festel GW Biofuels - economic aspects Chem Eng Technol 2008 31 5 715 720 10.1002/ceat.200700335

[B4] Girio FM Fonseca C Carvalheiro F Duarte LC Marques S Bogel-Lukasik R Hemicelluloses for fuel ethanol: a review Bioresour Technol 2010 101 13 4775 4800 10.1016/j.biortech.2010.01.088 20171088

[B5] Soccol CR Faraco V Karp S Vandenberghe LPS Thomaz-Soccol V Woiciechowski A Pandey A Pandey A, Larroche C, Ricke SC, Dussap CG, Gnansounou E lignocellulosic bioethanol: current status and future perspectives Biofuels: alternative feedstocks and conversion processes 2011 Oxford, UK: Elsevier Ltd 101 122

[B6] Sassner P Galbe M Zacchi G Techno-economic evaluation of bioethanol production from three different lignocellulosic materials Biomass Bioenergy 2008 32 5 422 430 10.1016/j.biombioe.2007.10.014

[B7] Jeffries TW Emerging technology for fermenting D-xylose Trends Biotechnol 1985 3 8 208 212 10.1016/0167-7799(85)90048-4

[B8] Kötter P Amore R Hollenberg CP Ciriacy M Isolation and characterization of the *Pichia stipitis* xylitol dehydrogenase gene, *XYL2*, and construction of a xylose-utilizing *Saccharomyces cerevisiae* transformant Curr Genet 1990 18 6 493 500 10.1007/BF00327019 2127555

[B9] Van Vleet JH Jeffries TW Yeast metabolic engineering for hemicellulosic ethanol production Curr Opin Biotechnol 2009 20 3 300 306 10.1016/j.copbio.2009.06.001 19545992

[B10] Hahn-Hägerdal B Karhumaa K Fonseca C Spencer-Martins I Gorwa-Grauslund MF Towards industrial pentose-fermenting yeast strains Appl Microbiol Biotechnol 2007 74 5 937 953 10.1007/s00253-006-0827-2 17294186

[B11] Camarasa C Faucet V Dequin S Role in anaerobiosis of the isoenzymes for *Saccharomyces cerevisiae* fumarate reductase encoded by *OSM1* and *FRDS1* Yeast 2007 24 5 391 401 10.1002/yea.1467 17345583

[B12] Arikawa Y Enomoto K Muratsubaki H Okazaki M Soluble fumarate reductase isoenzymes from *Saccharomyces cerevisiae* are required for anaerobic growth FEMS Microbiol Lett 1998 165 1 111 116 10.1111/j.1574-6968.1998.tb13134.x 9711846

[B13] Muratsubaki H Enomoto K One of the fumarate reductase isoenzymes from *Saccharomyces cerevisiae* is encoded by the *OSM1* gene Arch Biochem Biophys 1998 352 2 175 181 10.1006/abbi.1998.0583 9587404

[B14] Enomoto K Ohki R Muratsubaki H Cloning and sequencing of the gene encoding the soluble fumarate reductase from *Saccharomyces cerevisiae* DNA Res 1996 3 4 263 267 10.1093/dnares/3.4.263 8946166

[B15] Rossi C Hauber J Singer TP Mitochondrial and cytoplasmic enzymes for the reduction of fumarate to succinate in yeast Nature 1964 204 4954 167 170 10.1038/204167a0 14222265

[B16] Camarasa C Grivet JP Dequin S Investigation by ^13^C-NMR and tricarboxylic acid (TCA) deletion mutant analysis of pathways for succinate formation in *Saccharomyces cerevisiae* during anaerobic fermentation Microbiology 2003 149 2669 2678 10.1099/mic.0.26007-0 12949191

[B17] Enomoto K Arikawa Y Muratsubaki H Physiological role of soluble fumarate reductase in redox balancing during anaerobiosis in *Saccharomyces cerevisiae* FEMS Microbiol Lett 2002 215 1 103 108 10.1111/j.1574-6968.2002.tb11377.x 12393208

[B18] Frand AR Kaiser CA The *ERO1* gene of yeast is required for oxidation of protein dithiols in the endoplasmic reticulum Mol Cell 1998 1 2 161 170 10.1016/S1097-2765(00)80017-9 9659913

[B19] Pollard MG Travers KJ Weissman JS Ero1p: A novel and ubiquitous protein with an essential role in oxidative protein folding in the endoplasmic reticulum Mol Cell 1998 1 2 171 182 10.1016/S1097-2765(00)80018-0 9659914

[B20] Freedman RB Protein disulfide isomerase - multiple roles in the modification of nascent secretory proteins Cell 1989 57 7 1069 1072 10.1016/0092-8674(89)90043-3 2544299

[B21] Barlowe CK Miller EA Secretory protein biogenesis and traffic in the early secretory pathway Genetics 2013 193 2 383 410 10.1534/genetics.112.142810 23396477 PMC3567731

[B22] Frand AR Kaiser CA Ero1p oxidizes protein disulfide isomerase in a pathway for disulfide bond formation in the endoplasmic reticulum Mol Cell 1999 4 4 469 477 10.1016/S1097-2765(00)80198-7 10549279

[B23] Tu BP Ho-Schleyer SC Travers KJ Weissman JS Biochemical basis of oxidative protein folding in the endoplasmic reticulum Science 2000 290 5496 1571 1574 11090354 10.1126/science.290.5496.1571

[B24] Tu BP Weissman JS The FAD- and O_2_-dependent reaction cycle of Ero1-mediated oxidative protein folding in the endoplasmic reticulum Mol Cell 2002 10 5 983 994 10.1016/S1097-2765(02)00696-2 12453408

[B25] Bernales S Papa FR Walter P Intracellular signaling by the unfolded protein response Annu Rev Cell Dev Biol 2006 22 487 508 10.1146/annurev.cellbio.21.122303.120200 16822172

[B26] Travers KJ Patil CK Wodicka L Lockhart DJ Weissman JS Walter P Functional and genomic analyses reveal an essential coordination between the unfolded protein response and ER-associated degradation Cell 2000 101 3 249 258 10.1016/S0092-8674(00)80835-1 10847680

[B27] Runquist D Hahn-Hägerdal B Bettiga M Increased expression of the oxidative pentose phosphate pathway and gluconeogenesis in anaerobically growing xylose-utilizing *Saccharomyces cerevisiae* Microb Cell Fact 2009 8 49 10.1186/1475-2859-8-49 19778438 PMC2760498

[B28] Salusjärvi L Pitkänen JP Aristidou A Ruohonen L Penttilä M Transcription analysis of recombinant *Saccharomyces cerevisiae* reveals novel responses to xylose Appl Biochem Biotechnol 2006 128 3 237 261 10.1385/ABAB:128:3:237 16632884

[B29] Jin YS Laplaza JM Jeffries TW *Saccharomyces cerevisiae* engineered for xylose metabolism exhibits a respiratory response Appl Environ Microbiol 2004 70 11 6816 6825 10.1128/AEM.70.11.6816-6825.2004 15528549 PMC525251

[B30] Wahlbom CF Otero RRC van Zyl WH Hahn-Hägerdal B Jönsson LJ Molecular analysis of a *Saccharomyces cerevisiae* mutant with improved ability to utilize xylose shows enhanced expression of proteins involved in transport, initial xylose metabolism, and the pentose phosphate pathway Appl Environ Microbiol 2003 69 2 740 746 10.1128/AEM.69.2.740-746.2003 12570990 PMC143595

[B31] Bergdahl B Heer D Sauer U Hahn-Hägerdal B van Niel EW Dynamic metabolomics differentiates between carbon and energy starvation in recombinant *Saccharomyces cerevisiae* fermenting xylose Biotechnol Biofuels 2012 5 1 34 10.1186/1754-6834-5-34 22587303 PMC3462113

[B32] Mumberg D Muller R Funk M Yeast vectors for the controlled expression of heterologous proteins in different genetic backgrounds Gene 1995 156 1 119 122 10.1016/0378-1119(95)00037-7 7737504

[B33] Gietz RD Sugino A New yeast-*Escherichia coli* shuttle vectors constructed with *in vitro* mutagenized yeast genes lacking six-base pair restriction sites Gene 1988 74 2 527 534 10.1016/0378-1119(88)90185-0 3073106

[B34] Runquist D Fonseca C Rådstrom P Spencer-Martins I Hahn-Hägerdal B Expression of the Gxf1 transporter from *Candida intermedia* improves fermentation performance in recombinant xylose-utilizing *Saccharomyces cerevisiae* Appl Microbiol Biotechnol 2009 82 1 123 130 10.1007/s00253-008-1773-y 19002682

[B35] Runquist D Hahn-Hägerdal B Bettiga M Increased ethanol productivity in xylose-utilizing *Saccharomyces cerevisiae* via a randomly mutagenized xylose reductase Appl Environ Microbiol 2010 76 23 7796 7802 10.1128/AEM.01505-10 20889775 PMC2988607

[B36] Karhumaa K Hahn-Hägerdal B Gorwa-Grauslund MF Investigation of limiting metabolic steps in the utilization of xylose by recombinant *Saccharomyces cerevisiae* using metabolic engineering Yeast 2005 22 359 368 10.1002/yea.1216 15806613

[B37] Sambrook J Fritsch EF Maniatis T Molecular cloning: A laboratory manual 1989 Cold Spring Harbor Laboratory Press: Cold Spring Harbor

[B38] Gietz RD Schiestl RH High-efficiency yeast transformation using the LiAc/SS carrier DNA/PEG method Nat Protoc 2007 2 1 31 34 10.1038/nprot.2007.13 17401334

[B39] Gasch AP Spellman PT Kao CM Carmel-Harel O Eisen MB Storz G Botstein D Brown PO Genomic expression programs in the response of yeast cells to environmental changes Mol Biol Cell 2000 11 12 4241 4257 10.1091/mbc.11.12.4241 11102521 PMC15070

[B40] Roberts GG Hudson AP Transcriptome profiling of *Saccharomyces cerevisiae* during a transition from fermentative to glycerol-based respiratory growth reveals extensive metabolic and structural remodeling Mol Genet Genomics 2006 276 2 170 186 10.1007/s00438-006-0133-9 16741729

[B41] Rønnow B Kielland-Brandt MC *GUT2*, a gene for mitochondrial glycerol 3-phosphate dehydrogenase of *Saccharomyces cerevisiae* Yeast 1993 9 10 1121 1130 10.1002/yea.320091013 8256521

[B42] Grauslund M Rønnow B Carbon source-dependent transcriptional regulation of the mitochondrial glycerol-3-phosphate dehydrogenase gene, *GUT2*, from *Saccharomyces cerevisiae* Can J Microbiol 2000 46 12 1096 1100 10.1139/w00-105 11142398

[B43] Fleck CB Brock M Re-characterisation of *Saccharomyces cerevisiae* Ach1p: fungal CoA-transferases are involved in acetic acid detoxification Fungal Genet Biol 2009 46 6–7 473 485 19298859 10.1016/j.fgb.2009.03.004

[B44] Hahn-Hägerdal B Hallborn J Jeppsson H Meinander N Walfridsson M Ojamo H Penttilä M Zimmermann FK Asenjo JA, Andrews BA, Asenjo JA, Andrews BA Redox balances in recombinant *Saccharomyces cerevisiae* Ann N Y Acad Sci, vol. 782 1996 New York: New York Acad Sciences 286 296 10.1111/j.1749-6632.1996.tb40569.x 8659905

[B45] Wahlbom CF Hahn-Hägerdal B Furfural, 5-hydroxymethyl furfural, and acetoin act as external electron acceptors during anaerobic fermentation of xylose in recombinant *Saccharomyces cerevisiae* Biotechnol Bioeng 2002 78 2 172 178 10.1002/bit.10188 11870608

[B46] Sonderegger M Jeppsson M Hahn-Hägerdal B Sauer U Molecular basis for anaerobic growth of *Saccharomyces cerevisiae* on xylose, investigated by global gene expression and Metabolic Flux Analysis Appl Environ Microbiol 2004 70 4 2307 2317 10.1128/AEM.70.4.2307-2317.2004 15066826 PMC383160

[B47] Salusjärvi L Kaunisto S Holmström S Vehkomäki M-L Koivuranta K Pitkänen J-P Ruohonen L Overexpression of NADH-dependent fumarate reductase improves D-xylose fermentation in recombinant *Saccharomyces cerevisiae* J Ind Microbiol Biotechnol 2013 40 1383 1392 10.1007/s10295-013-1344-9 24113892

[B48] Bradley PH Brauer MJ Rabinowitz JD Troyanskaya OG Coordinated concentration changes of transcripts and metabolites in *Saccharomyces cerevisiae* PLoS Comput Biol 2009 5 1 e1000270 10.1371/journal.pcbi.1000270 19180179 PMC2614473

[B49] Abdulrehman D Monteiro PT Teixeira MC Mira NP Lourenço AB dos Santos SC Cabrito TR Francisco AP Madeira SC Aires RS Oliveira AL Sá-Correia I Freitas AT YEASTRACT: providing a programmatic access to curated transcriptional regulatory associations in *Saccharomyces cerevisiae* through a web services interface Nucleic Acids Res 2011 39 suppl 1 D136 D140 20972212 10.1093/nar/gkq964 PMC3013800

[B50] Herrero E Ros J Bellí G Cabiscol E Redox control and oxidative stress in yeast cells BBA General Subjects 2008 1780 11 1217 1235 10.1016/j.bbagen.2007.12.004 18178164

[B51] Gross E Sevier CS Heldman N Vitu E Bentzur M Kaiser CA Thorpe C Fass D Generating disulfides enzymatically: Reaction products and electron acceptors of the endoplasmic reticulum thiol oxidase Ero1p Proc Natl Acad Sci U S A 2006 103 2 299 304 10.1073/pnas.0506448103 16407158 PMC1326156

[B52] Tzagoloff A Jang J Glerum DM Wu M *FLX1* codes for a carrier protein involved in maintaining a proper balance of flavin nucleotides in yeast mitochondria J Biol Chem 1996 271 13 7392 7397 10.1074/jbc.271.13.7392 8631763

[B53] Protchenko O Rodriguez-Suarez R Androphy R Bussey H Philpott CC A screen for genes of heme uptake identifies the *FLC* family required for import of FAD into the endoplasmic reticulum J Biol Chem 2006 281 30 21445 21457 10.1074/jbc.M512812200 16717099

[B54] Farquhar R Honey N Murant SJ Bossier P Schultz L Montgomery D Ellis RW Freedman RB Tuite MF Protein disulfide isomerase is essential for viability in *Saccharomyces cerevisiae* Gene 1991 108 1 81 89 10.1016/0378-1119(91)90490-3 1761235

[B55] Karhumaa K Påhlman A-K Hahn-Hägerdal B Levander F Gorwa-Grauslund M-F Proteome analysis of the xylose-fermenting mutant yeast strain TMB3400 Yeast 2009 26 7 371 382 10.1002/yea.1673 19504622

[B56] Kim S Sideris DP Sevier CS Kaiser CA Balanced Ero1 activation and inactivation establishes ER redox homeostasis J Cell Biol 2012 196 6 713 725 10.1083/jcb.201110090 22412017 PMC3308690

[B57] Laboissiere MCA Sturley SL Raines RT The essential function of protein-disulfide isomerase is to unscramble nonnative disulfide bonds J Biol Chem 1995 270 47 28006 28009 10.1074/jbc.270.47.28006 7499282

[B58] Gauss R Kanehara K Carvalho P Ng DTW Aebi M A complex of Pdi1p and the mannosidase Htm1p initiates clearance of unfolded glycoproteins from the endoplasmic reticulum Mol Cell 2011 42 6 782 793 10.1016/j.molcel.2011.04.027 21700223

