# Supplementary material for: Physiological effects of over-expressing compartment-specific components of the protein folding machinery in xylose-fermenting Saccharomyces cerevisiae
Source: BMC Biotechnol. 2014 Apr 23;14:28. doi: 10.1186/1472-6750-14-28 (PMC4021093; doi:10.1186/1472-6750-14-28)
Supplement: Additional file 1 — Supplementary Figures S1-S5. Description: FR activity measurements, DTT sensitivity results, fermentation profiles. [file 1472-6750-14-28-S1.pdf]

ADDITIONAL FILE 1

Physiological effects of over-expressing compartment-specific components of the protein folding machinery in xylose-fermenting *Saccharomyces cerevisiae*

Basti Bergdahl, Marie F. Gorwa-Grauslund and Ed W.J. van Niel

Division of Applied Microbiology, Department of Chemistry, Lund University, P.O. Box 124, SE-22100 Lund, Sweden

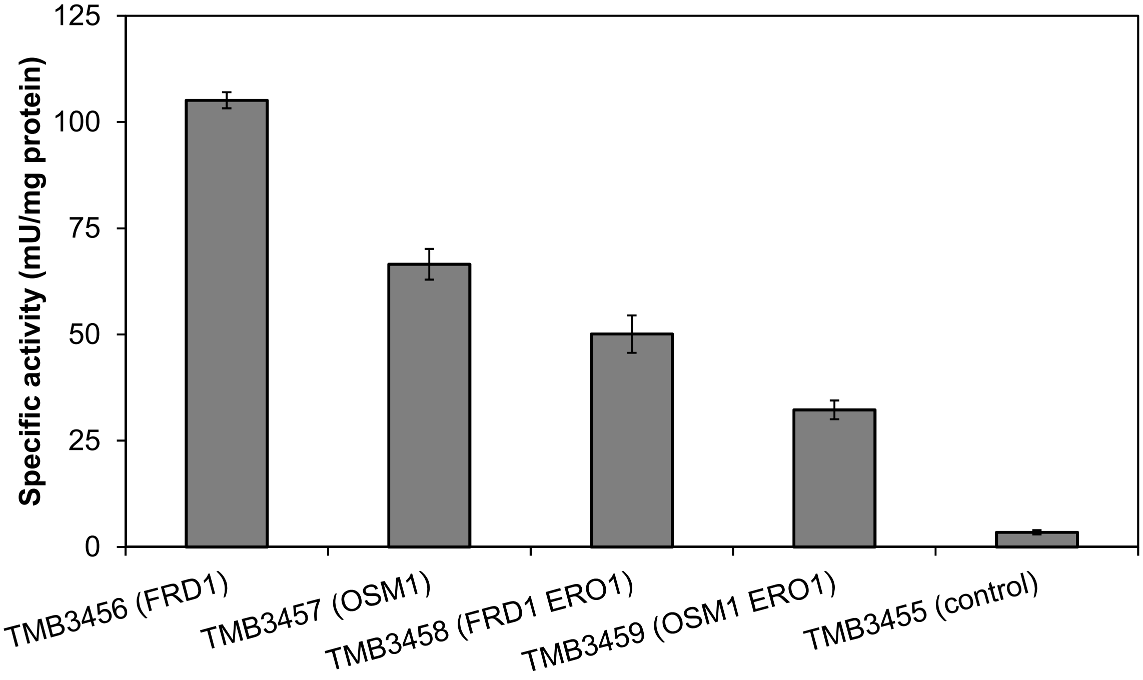

Figure S1. Specific activity of fumarate reductase on xylose.
The specific activity was determined in cell-free protein extracts of cells grown anaerobically in 2X YNB medium containing glucose and xylose as carbon source. The cells were harvested after 36 h when xylose was the only carbon source. Data represent mean ± standard deviation of two independent extracts.

TMB3455(ctrl) TMB3456(*FRD1*) TMB3457(*OSM1*)

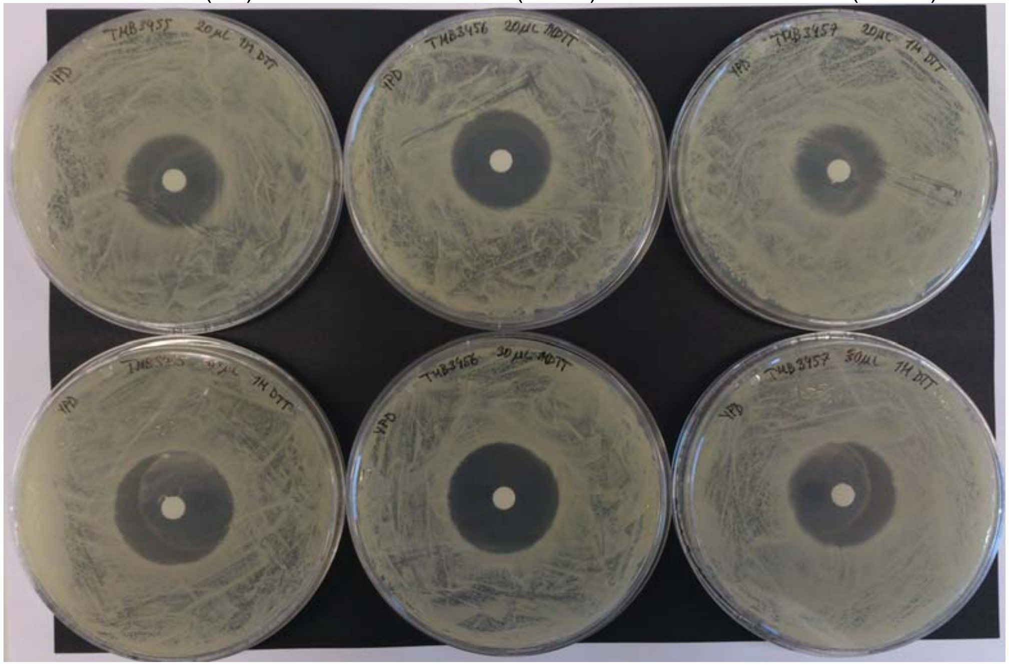

20 µL 1M DTT

30 µL 1M DTT

TMB3455(ctrl) TMB3458(*FRD1 ERO1*) TMB3459(*OSM1 ERO1*)

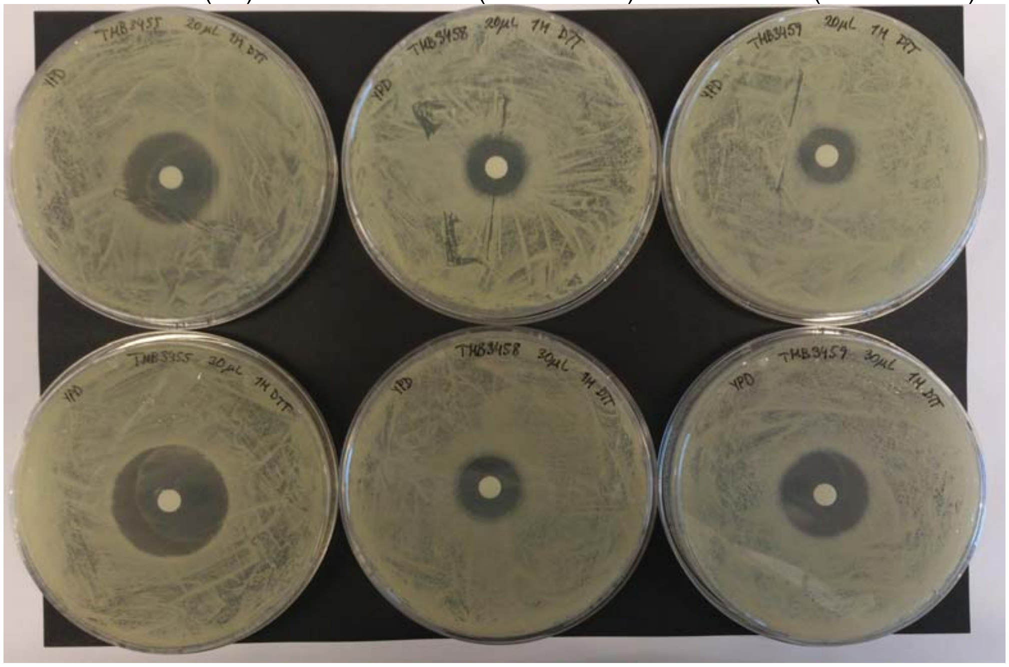

20 µL 1M DTT

30 µL 1M DTT

Figure S2. DTT sensitivity test.
Exponentially growing cells (2×10^6 cells) were spread on solid YPD medium and exposed to 20 µL or 30 µL of 1M DTT. After 48 h incubation at 30°C the diameters of the halos around the discs were measured. The following results were obtained using 30 µL 1M DTT (mean±SD in mm): TMB3455, 33.5±2.1; TMB3456, 31.0±0.0; TMB3457, 30.5±2.1; TMB3458, 22.5±6.4; TMB3459, 26±2.8. The strains over-expressing *ERO1* showed much smaller halo than the other strains demonstrating a higher activity.

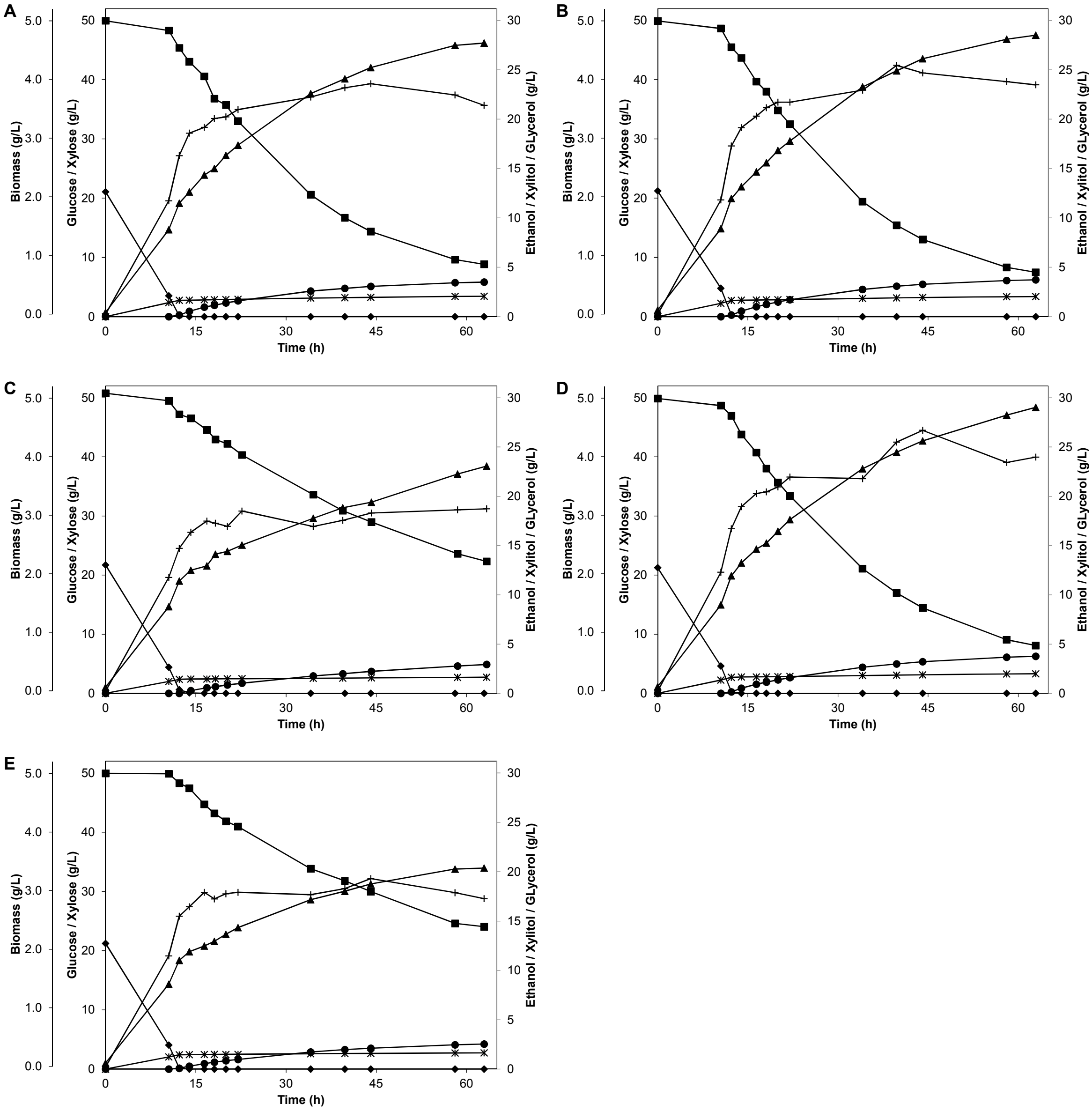

Figure S3. Fermentation of a glucose/xylose mix in complex medium.
A mix of 20 g/L glucose and 50 g/L xylose was fermented in complex medium (10 g/L yeast extract) by strains **A)** TMB3455 (control), **B)** TMB3456 (*FRD1*), **C)** TMB3457 (*OSM1*), **D)** TMB3458 (*FRD1 ERO1*) and **E)** TMB3459 (*OSM1 ERO1*). Symbols: diamonds, glucose; squares, xylose; plus, biomass; triangles, ethanol; circles, xylitol; stars, glycerol. Figures illustrate one representative experiment out of two biological replicates.

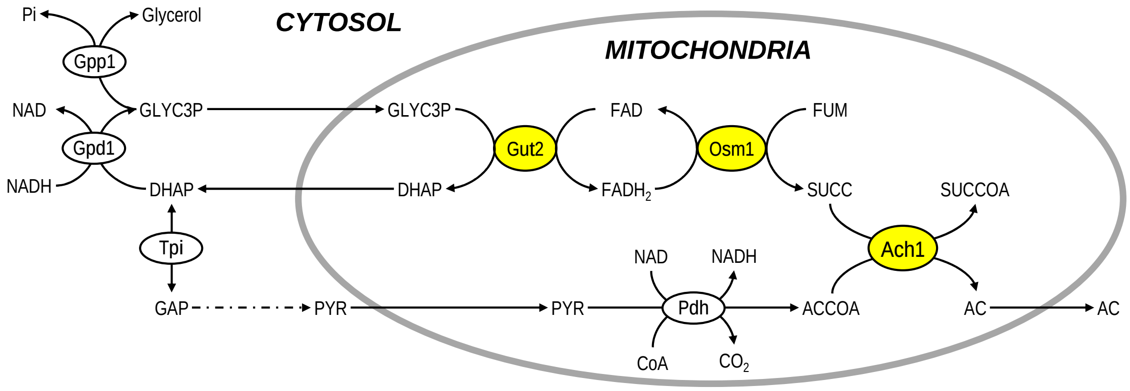

Figure S4. The Gut2p/Osm1p redox-couple during xylose fermentation
During xylose fermentation several genes related to respiratory metabolism are derepressed, including *GUT2* which encodes a FAD-dependent glycerol 3-phosphate dehydrogenase and *ACH1* encoding a coenzyme A transferase. When combined with Osm1p (the mitochondrial fumarate reductase) carbon may be diverted from glycerol to acetate at the expense of acetyl-CoA needed for biosynthesis.

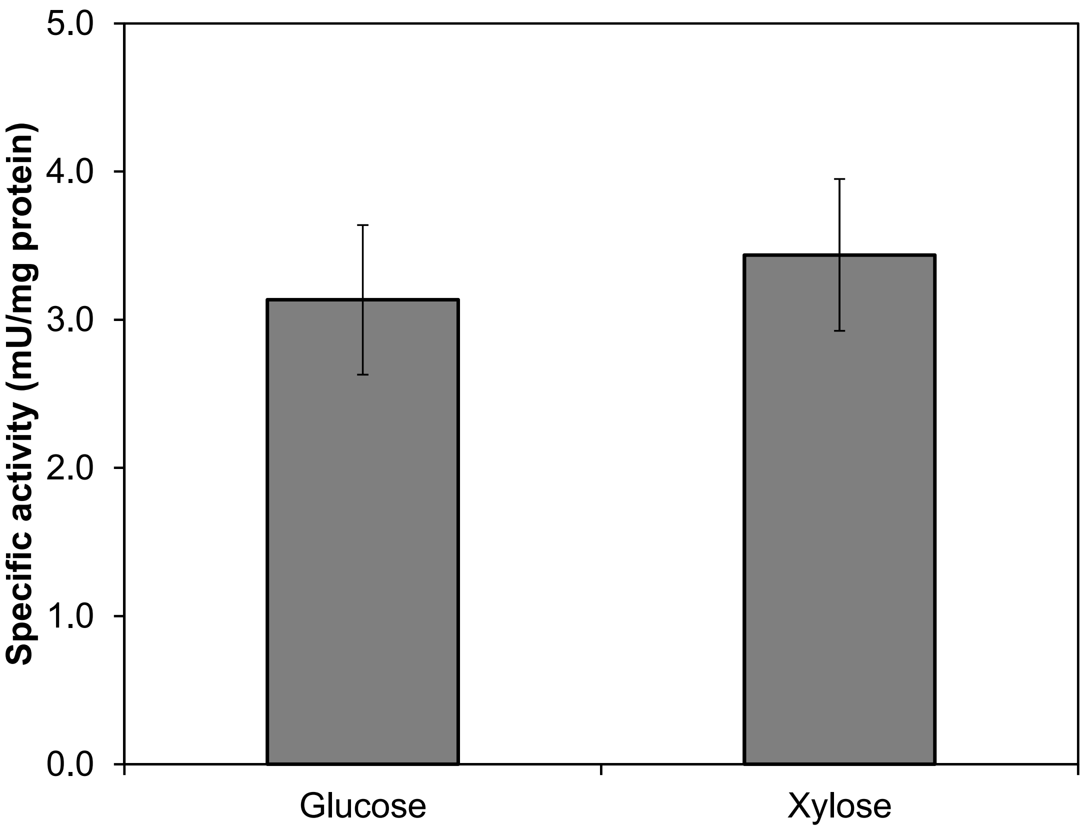

Figure S5. Specific activity of fumarate reductase in TMB3455 on glucose and xylose.
The specific activity was determined in cell-free protein extracts of cells grown anaerobically in 2X YNB medium containing glucose and xylose as carbon source. The cells were harvested after 12 h during growth on glucose or after 36 h when xylose was the only carbon source. Data represent mean ± standard deviation of two independent extracts.
