# Supplementary material for: Physiological effects of over-expressing compartment-specific components of the protein folding machinery in xylose-fermenting Saccharomyces cerevisiae
Source: BMC Biotechnol. 2014 Apr 23;14:28. doi: 10.1186/1472-6750-14-28 (PMC4021093; doi:10.1186/1472-6750-14-28)
Supplement: Additional file 2 — Supplementary Tables S1-S5. Description: Genes included in the cluster analysis, maximum specific growth rates and product yields using strains TMB3455, TMB3456 and TMB3458. [file 1472-6750-14-28-S2.pdf]

ADDITIONAL FILE 2

Physiological effects of over-expressing compartment-specific components of the protein folding machinery in xylose-fermenting *Saccharomyces cerevisiae*

Basti Bergdahl, Marie F. Gorwa-Grauslund and Ed W.J. van Niel

Division of Applied Microbiology, Department of Chemistry, Lund University, P.O. Box 124, SE-22100 Lund, Sweden

Table S1. Carbon and degree of reduction balances for the glucose and xylose phases in defined and complex media. Values are given as mean ± standard deviation of two independent experiments.

	Defined medium				Complex medium			
	Glucose		Xylose		Glucose		Xylose	
	Carbon balance (%)*	Redox balance (%)	Carbon balance (%)*	Redox balance (%)	Carbon balance (%)*	Redox balance (%)	Carbon balance (%)*	Redox balance (%)
**TMB3455 (control)**	107±2	112±3	89±3	95±3	129±21	158±29	103±8	109±8
**TMB3456 (*FRD1*)**	119±1	127±1	81±4	86±4	145±10	162±12	101±3	107±3
**TMB3457 (*OSM1*)**	111±9	118±12	100±9	107±9	134±5	149±8	105±3	111±3
**TMB3458 (*FRD1 ERO1*)**	114±11	122±12	81±5	86±6	147±12	167±21	94±10	99±10
**TMB3459 (*OSM1 ERO1*)**	114±16	119±22	75±2	80±3	138±7	150±11	83±3	88±3

* The specific production rate of CO_2 was estimated according to the following equation:
r_{CO_2} (mmol/g CDW/h) = $r_{etoh} + r_{ac}$ + 0.1 mol CO_2/C-mol biomass × 1 C-mol biomass/24.6 g CDW × μ_{max}

Table S2. Transcription data used for hierarchical cluster analysis. Values are given as the mean log_2 fold change ± pooled standard deviation of two independent measurements in each condition. P-values from a two-tailed student's t-test assuming equal variance are given within parenthesis.

		Anaerobic vs. aerobic		Xylose vs. glucose
**Gene**	**Process**	**Glucose**	**Xylose**	**Anaerobic**
EPT1	LIPID/INOSITOL METABOLISM<br>Phospholipid biosynthesis	0.66±0.05 (0.01)	-0.43±0.09 (0.04)	-1.66±0.04 (0.00)
ALG7	GLYCOSYLATION/MODIFICATION<br>Glycoprotein processing	-0.03±0.12 (0.81)	-1.36±0.20 (0.02)	-1.61±0.18 (0.01)
DFR1	VITAMINS BIOSYNTHESIS<br>Folate biosynthesis	-0.23±0.11 (0.18)	-1.35±0.05 (0.00)	-1.30±0.04 (0.00)
SWP1	GLYCOSYLATION/MODIFICATION<br>Oligosaccharyltransferase	-0.07±0.16 (0.71)	-1.24±0.21 (0.03)	-1.40±0.25 (0.03)
MPD1	PROTEIN FOLDING<br>Disulfide bond formation	0.21±0.15 (0.29)	-0.98±0.26 (0.07)	-1.34±0.28 (0.04)
SEC72	TRANSLOCATION<br>Posttranslational translocation	0.16±0.16 (0.43)	-0.81±0.21 (0.06)	-1.27±0.26 (0.04)
MCD4	GLYCOSYLATION/MODIFICATION<br>GPI anchoring	0.22±0.16 (0.30)	-0.73±0.16 (0.04)	-1.06±0.22 (0.04)
SFB2	VESICLE TRAFFICKING/TRANSPORT<br>Budding (ER-Golgi)	0.21±0.23 (0.46)	-0.68±0.12 (0.03)	-0.93±0.24 (0.06)
LHS1	PROTEIN FOLDING<br>Chaperones	0.12±0.12 (0.42)	-0.70±0.15 (0.04)	-0.92±0.13 (0.02)
SEC12	VESICLE TRAFFICKING/TRANSPORT<br>Budding (ER-Golgi)	0.12±0.09 (0.35)	-0.56±0.01 (0.00)	-0.79±0.01 (0.00)
OST3	GLYCOSYLATION/MODIFICATION<br>Oligosaccharyltransferase	0.03±0.06 (0.65)	-0.51±0.03 (0.00)	-0.75±0.06 (0.01)
SEC59	GLYCOSYLATION/MODIFICATION: Protein<br>N-glycosylation	0.07±0.09 (0.51)	-0.54±0.01 (0.00)	-0.97±0.08 (0.01)
SEC71 (*SEC66*)	TRANSLOCATION<br>Posttranslational translocation	0.06±0.14 (0.69)	-0.58±0.15 (0.06)	-0.91±0.20 (0.04)
ERD2	VESICLE TRAFFICKING/TRANSPORT<br>Retrieval (Golgi-ER)	-0.01±0.06 (0.89)	-0.67±0.04 (0.00)	-0.91±0.06 (0.00)
MPD2	PROTEIN FOLDING<br>Disulfide bond formation	0.00±0.24 (0.99)	-0.85±0.27 (0.09)	-0.79±0.33 (0.14)
SPC2	TRANSLOCATION<br>Signal peptide processing	-0.09±0.12 (0.55)	-0.76±0.22 (0.07)	-0.66±0.23 (0.10)
RAM2	GLYCOSYLATION/MODIFICATION<br>Glycoprotein processing	-0.17±0.19 (0.48)	-0.75±0.11 (0.02)	-0.76±0.21 (0.07)
BFR1	VESICLE TRAFFICKING/TRANSPORT<br>Distal secretion	-0.08±0.07 (0.39)	-0.63±0.14 (0.05)	-0.72±0.16 (0.04)
MNN11	GLYCOSYLATION/MODIFICATION<br>Golgi/O-linked glycosylation	-0.05±0.06 (0.49)	-0.63±0.10 (0.02)	-0.82±0.03 (0.00)
STE24	GLYCOSYLATION/MODIFICATION<br>Glycoprotein processing	0.11±0.04 (0.13)	-0.36±0.10 (0.08)	-0.82±0.11 (0.02)
GAA1	GLYCOSYLATION/MODIFICATION<br>GPI anchoring	0.19±0.12 (0.24)	-0.37±0.05 (0.02)	-0.72±0.11 (0.02)
SEC13	VESICLE TRAFFICKING/TRANSPORT<br>Budding (ER-Golgi)	0.11±0.02 (0.04)	-0.35±0.08 (0.05)	-0.61±0.07 (0.01)
WBP1	GLYCOSYLATION/MODIFICATION<br>Oligosaccharyltransferase	-0.03±0.12 (0.85)	-0.51±0.11 (0.05)	-0.63±0.16 (0.06)
ECM31	VITAMINS BIOSYNTHESIS<br>Pantothenate biosynthesis	-0.05±0.11 (0.67)	-0.52±0.12 (0.05)	-0.52±0.12 (0.05)
TRS120	VESICLE TRAFFICKING/TRANSPORT<br>Fusion (ER-Golgi)	-0.04±0.03 (0.36)	-0.59±0.13 (0.05)	-0.20±0.13 (0.27)
ERV25	VESICLE TRAFFICKING/TRANSPORT<br>Budding (ER-Golgi)	-0.08±0.16 (0.66)	-0.45±0.09 (0.04)	-0.43±0.17 (0.13)
VPS54	VACUOLAR PROTEIN SORTING	0.02±0.15 (0.93)	-0.40±0.05 (0.02)	-0.40±0.16 (0.12)
ACB1	LIPID/INOSITOL METABOLISM<br>Fatty acid metabolism	0.09±0.12 (0.54)	-0.42±0.14 (0.10)	-0.29±0.17 (0.24)

SCS3	LIPID/INOSITOL METABOLISM<br>Phospholipid biosynthesis	0.16±0.10 (0.24)	-0.30±0.10 (0.10)	-0.40±0.10 (0.06)
BOS1	VESICLE TRAFFICKING/TRANSPORT<br>Fusion (ER-Golgi)	-0.08±0.13 (0.60)	-0.23±0.04 (0.03)	-0.26±0.13 (0.18)
ERO1	PROTEIN FOLDING<br>Disulfide bond formation	0.02±0.06 (0.74)	0.00±0.04 (0.99)	-0.50±0.04 (0.01)
JEM1	PROTEIN FOLDING<br>Chaperones	0.34±0.16 (0.16)	0.02±0.18 (0.93)	-0.62±0.10 (0.03)
YPT10	VESICLE TRAFFICKING/TRANSPORT<br>Distal secretion	0.53±0.14 (0.07)	0.35±0.03 (0.01)	-0.25±0.02 (0.00)
KTR1	GLYCOSYLATION/MODIFICATION<br>Golgi/O-linked glycosylation	0.53±0.06 (0.01)	0.58±0.02 (0.00)	-0.22±0.04 (0.03)
SFB3	VESICLE TRAFFICKING/TRANSPORT<br>Budding (ER-Golgi)	0.47±0.20 (0.15)	0.62±0.19 (0.08)	0.00±0.19 (0.99)
VPS35	VACUOLAR PROTEIN SORTING	0.25±0.19 (0.32)	0.51±0.12 (0.05)	0.01±0.19 (0.98)
MNS1	GLYCOSYLATION/MODIFICATION<br>Glycoprotein processing	0.37±0.47 (0.51)	1.12±0.34 (0.08)	-0.23±0.57 (0.73)
PDI1	PROTEIN FOLDING<br>Disulfide bond formation	0.54±0.26 (0.17)	1.31±0.31 (0.05)	-0.06±0.08 (0.55)
TUS1	VESICLE TRAFFICKING/TRANSPORT<br>Distal secretion	0.19±0.05 (0.06)	0.46±0.07 (0.02)	0.42±0.07 (0.03)
PEX4	PROTEIN DEGRADATION<br>Ubiquitin/proteasome	-0.04±0.09 (0.68)	0.73±0.23 (0.08)	0.56±0.04 (0.00)
DOA4	PROTEIN DEGRADATION<br>Ubiquitin/proteasome	0.04±0.12 (0.80)	0.55±0.08 (0.02)	0.82±0.14 (0.03)
RIB1	VITAMINS BIOSYNTHESIS<br>Flavin biosynthesis	0.15±0.20 (0.53)	1.07±0.32 (0.08)	1.38±0.25 (0.03)
ECM8	CELL WALL BIOGENESIS	-0.34±0.12 (0.11)	1.69±0.44 (0.06)	1.65±0.44 (0.06)
YET2	VESICLE TRAFFICKING/TRANSPORT<br>Budding (ER-Golgi)	-0.11±0.36 (0.79)	2.14±0.27 (0.02)	2.68±0.41 (0.02)
ECM3	CELL WALL BIOGENESIS	0.81±0.01 (0.00)	2.95±0.08 (0.00)	0.83±0.06 (0.00)
HEM13	PORPHYRIN COMPOUNDS BIOSYNTHESIS<br>Heme biosynthesis	3.18±0.13 (0.00)	2.46±0.11 (0.00)	-0.14±0.10 (0.32)
ARE1	LIPID/INOSITOL METABOLISM<br>Sterol metabolism	2.21±0.38 (0.03)	2.80±0.25 (0.01)	-0.64±0.40 (0.25)
CSR1	CELL WALL BIOGENESIS	2.12±0.45 (0.04)	2.58±0.35 (0.02)	-0.84±0.52 (0.25)
PMT5	GLYCOSYLATION/MODIFICATION<br>Golgi/O-linked glycosylation	1.89±0.30 (0.02)	2.05±0.15 (0.01)	-0.30±0.31 (0.45)
PMT3	GLYCOSYLATION/MODIFICATION<br>Golgi/O-linked glycosylation	1.75±0.38 (0.04)	1.96±0.25 (0.02)	-0.24±0.43 (0.63)
HAP1	TRANSCRIPTION FACTOR	1.65±0.41 (0.06)	2.09±0.30 (0.02)	0.38±0.50 (0.52)
MGA2	LIPID/INOSITOL METABOLISM<br>Fatty acid metabolism	1.84±0.11 (0.00)	1.13±0.07 (0.00)	0.26±0.11 (0.14)
HMG2	LIPID/INOSITOL METABOLISM<br>Sterol metabolism	1.60±0.11 (0.00)	1.23±0.21 (0.03)	-0.57±0.06 (0.01)
EUG1	PROTEIN FOLDING<br>Disulfide bond formation	1.65±0.11 (0.00)	1.31±0.26 (0.04)	-1.47±0.28 (0.03)

Table S3. Maximum specific growth rates and product yields in anaerobic batch fermentation of 20 g/L glucose and 50 g/L xylose in defined medium (2X YNB) using strains TMB3455, TMB3456 and TMB3458. Values are given as mean ± standard deviation of two independent experiments.

	Glucose phase			Xylose phase		
	**TMB3455 (control)**	**TMB3456 (*FRD1*)**	**TMB3458 (*FRD1 ERO1*)**	**TMB3455 (control)**	**TMB3456 (*FRD1*)**	**TMB3458 (*FRD1 ERO1*)**
μ_{max} (1/h)	0.29±0.012	0.33±0.028	0.30±0.029	0.029±0.002	0.023±0.006	0.029±0.005
**Yields per substrate (mol/mol sugar)**						
$Y_{xylt/s}$	–	–	–	0.24±0.03	0.20±0.01	0.22±0.03
$Y_{glyc/s}$	0.22±0.02	0.24±0.02	0.23±0.02	0.020±0.000	0.021±0.002	0.017±0.002
$Y_{ac/s}$	0.020±0.001	0.020±0.004	0.019±0.005	0.019±0.001	0.018±0.005	0.023±0.002
$Y_{etoh/s}$	1.87±0.14	2.08±0.06	2.00±0.20	1.03±0.00	0.97±0.03	0.93±0.04
$Y_{succ/s}$	0.003±0.000	0.002±0.001	0.003±0.001	0.009±0.000	0.010±0.002	0.007±0.002
$Y_{x/s}$ (g/mol)	10.4±0.5	11.7±0.7	10.7±1.1	3.8±0.4	2.9±1.1	3.8±0.2
**Yields per biomass (mmol/g CDW)**						
$Y_{xylt/x}$	–	–	–	63.1±0.3	73.1±22.4	57.9±9.8
$Y_{glyc/x}$	20.2±0.8	18.9±0.3	18.9±1.6	5.2±0.6	7.6±1.8	4.4±0.2
$Y_{ac/x}$	2.1±0.1	1.8±0.1	1.9±0.4	5.1±0.3	6.3±0.6	6.0±0.8
$Y_{etoh/x}$	153±8	140±4	151±1	271±30	356±124	243±24
$Y_{succ/x}$	0.19±0.04	0.15±0.01	0.26±0.06	2.5±0.4	3.7±0.9	1.3±0.5

Table S4. Maximum specific growth rates and product yields in anaerobic batch fermentation of 20 g/L glucose and 50 g/L xylose in complex medium (10 g/L yeast extract) using strains TMB3455, TMB3456 and TMB3458. Values are given as mean ± standard deviation of two independent experiments.

	Glucose phase			Xylose phase		
	**TMB3455 (control)**	**TMB3456 (*FRD1*)**	**TMB3458 (*FRD1 ERO1*)**	**TMB3455 (control)**	**TMB3456 (*FRD1*)**	**TMB3458 (*FRD1 ERO1*)**
μ_{max} (1/h)	0.37±0.003	0.37±0.000	0.38±0.004	0.033±0.004	0.030±0.006	0.035±0.008
**Yields per substrate (mol/mol sugar)**						
$Y_{xylt/s}$	–	–	–	0.14±0.01	0.16±0.01	0.14±0.02
$Y_{glyc/s}$	0.13±0.01	0.10±0.00	0.11±0.00	0.013±0.001	0.014±0.003	0.011±0.003
$Y_{ac/s}$	0.030±0.006	0.019±0.000	0.022±0.001	0.022±0.003	0.023±0.001	0.024±0.002
$Y_{etoh/s}$	2.7±0.6	2.8±0.3	2.9±0.5	1.4±0.1	1.4±0.1	1.3±0.1
$Y_{succ/s}$	0.005±0.004	0.002±0.000	0.003±0.001	0.003±0.001	0.004±0.000	0.004±0.000
$Y_{x/s}$ (g/mol)	17.5±1.4	19.3±0.5	18.3±2.2	6.4±0.8	6.0±0.2	6.7±0.3
**Yields per biomass (mmol/g CDW)**						
$Y_{xylt/x}$	–	–	–	22.3±0.8	26.4±2.3	20.6±2.3
$Y_{glyc/x}$	7.3±0.5	6.9±0.6	6.8±0.3	2.1±0.0	2.4±0.6	1.7±0.3
$Y_{ac/x}$	1.3±0.4	1.4±0.2	1.3±0.0	3.4±0.0	3.9±0.1	3.5±0.2
$Y_{etoh/x}$	94.2±5.0	92.0±6.9	93.2±6.0	222±11	230±6	189±9
$Y_{succ/x}$	0.12±0.07	0.20±0.09	0.15±0.000	0.54±0.03	0.62±0.03	0.55±0.03

Table S5. Transcription data from Runquist *et al*. (Microb Cell Fact 2009, 8) that is referred to in the main text. Values are given as the mean $\log_2$ fold change ± pooled standard deviation of two independent measurements in each condition. *P*-values from a two-tailed student's t-test assuming equal variance are given within parenthesis.

Gene	Description	Anaerobic vs. aerobic		Xylose vs. glucose	
		Glucose	Xylose	Anaerobic	Aerobic
KAR2	ATPase acting as a chaperone to mediate protein folding in the ER; regulates the unfolded protein response via interaction with Ire1p	0.53±0.21 (0.13)	0.60±0.25 (0.14)	-0.45±0.20 (0.16)	-0.51±0.26 (0.19)
INO1	Inositol 1-phosphate synthase, involved in synthesis of inositol phosphates and inositol-containing phospholipids	1.68±0.44 (0.06)	-0.20±0.75 (0.81)	2.74±0.85 (0.08)	4.62±0.15 (0.00)
ERO1	Thiol oxidase required for oxidative protein folding in the endoplasmic reticulum	0.02±0.06 (0.74)	0.00±0.04 (0.99)	-0.50±0.04 (0.01)	-0.47±0.06 (0.02)
MDH2	Cytoplasmic malate dehydrogenase	-0.39±0.22 (0.22)	0.80±0.17 (0.04)	1.90±0.20 (0.01)	0.71±0.20 (0.07)
GUT2	Mitochondrial glycerol-3-phosphate dehydrogenase	-0.61±0.06 (0.01)	-0.04±0.51 (0.94)	2.92±0.51 (0.03)	2.35±0.07 (0.00)
ACH1	Coenzyme A transferase, primarily localized to mitochondria	0.43±0.45 (0.44)	0.27±0.53 (0.67)	2.24±0.67 (0.08)	2.40±0.17 (0.00)
MSN4	Transcriptional activator related to Msn2p; activated in stress conditions, which results in translocation from the cytoplasm to the nucleus	0.12±0.21 (0.62)	1.25±0.17 (0.02)	2.05±0.24 (0.01)	0.93±0.13 (0.02)
YAP1	Basic leucine zipper (bZIP) transcription factor required for oxidative stress tolerance	0.01±0.12 (0.92)	0.46±0.08 (0.03)	0.70±0.12 (0.03)	0.25±0.08 (0.09)
PDI1	Protein disulfide isomerase, multifunctional protein resident in the ER, essential for the formation of disulfide bonds in secretory and cell-surface proteins	0.54±0.26 (0.17)	1.31±0.31 (0.05)	-0.06±0.08 (0.55)	-0.83±0.39 (0.17)
